# A Novel Workflow for In Silico Prediction of Bioactive Peptides: An Exploration of *Solanum lycopersicum* By-Products

**DOI:** 10.3390/biom14080930

**Published:** 2024-07-31

**Authors:** Francesco Morena, Chiara Cencini, Eleonora Calzoni, Sabata Martino, Carla Emiliani

**Affiliations:** 1 Section of Biochemistry and Molecular Biology, Department of Chemistry, Biology and Biotechnology, University of Perugia, Via del Giochetto, 06123 Perugia, Italy; chiara.cencini@studenti.unipg.it (C.C.); eleonora.calzoni@unipg.it (E.C.); sabata.martino@unipg.it (S.M.); 2 Centro di Eccellenza su Materiali Innovativi Nanostrutturati (CEMIN), University of Perugia, Via del Giochetto, 06123 Perugia, Italy

**Keywords:** in silico prediction, bioactive peptides, food-derived peptides, tomato bioactive compounds, plant-derived peptides, health-promoting peptides, functional foods, nutritional genomics

## Abstract

Resource-intensive processes currently hamper the discovery of bioactive peptides (BAPs) from food by-products. To streamline this process, in silico approaches present a promising alternative. This study presents a novel computational workflow to predict peptide release, bioactivity, and bioavailability, significantly accelerating BAP discovery. The computational flowchart has been designed to identify and optimize critical enzymes involved in protein hydrolysis but also incorporates multi-enzyme screening. This feature is crucial for identifying the most effective enzyme combinations that yield the highest abundance of BAPs across different bioactive classes (anticancer, antidiabetic, antihypertensive, anti-inflammatory, and antimicrobial). Our process can be modulated to extract diverse BAP types efficiently from the same source. Here, we show the potentiality of our method for the identification of diverse types of BAPs from by-products generated from *Solanum lycopersicum*, the widely cultivated tomato plant, whose industrial processing generates a huge amount of waste, especially tomato peel. In particular, we optimized tomato by-products for bioactive peptide production by selecting cultivars like Line27859 and integrating large-scale gene expression. By integrating these advanced methods, we can maximize the value of by-products, contributing to a more circular and eco-friendly production process while advancing the development of valuable bioactive compounds.

## 1. Introduction

Over the past few years, the production, characterization, and application of protein hydrolysates have sparked significant interest. This is primarily due to their potential benefits and functional properties, which hold promise for both health and industry [1,2,3,4,5,6,7,8].

Protein hydrolysates, derived from the enzymatic or chemical breakdown of proteins, are a diverse blend of oligopeptides ranging from 3 to 50 amino acid residues in length along with free amino acids [1,2,3,4,5,6,7,8,9]. Their amino acid composition and sequences significantly influence their bioactivity; these peptides have been found to exhibit a wide range of biological functions, including antioxidant [10,11], antimicrobial [12,13], immunomodulatory [14,15], hypotensive [16], antitumor [17,18], anticoagulant [19], hypoglycemic [20], and cholesterol-lowering effects [21]. The effectiveness of biopeptides is primarily attributed to their bioavailability [22]. To be active, biopeptides must be resistant to degradation by gastrointestinal proteases and brush-border peptidases, allowing them to be absorbed through the intestinal epithelium and reach the bloodstream in an active form, thereby exerting their effects [23,24].

Protein hydrolysates can be cost-effectively produced from protein-rich by-products, especially food by-products. Food processing industries typically generate significant amounts of by-products with high protein contents, often discarded or processed into low-value protein-based products. In this regard, tomatoes are an ideal source of protein-rich by-products. Tomatoes are a widely consumed vegetable crop worldwide, rich in micronutrients and other bioactive compounds essential or beneficial for human health [25,26,27]. The wastes generated by the tomato processing industry, particularly peels—estimated at 180 million tons per year—represent a great source of bioactive compounds [28,29], which have the potential to contribute to a circular economy aimed at reducing food waste [30].

Enzymatic hydrolysis of protein-rich food by-products offers an effective method for producing protein hydrolysates containing various bioactive peptides (BAPs), depending on the enzymes’ specificities and the hydrolysis conditions [31,32,33,34,35]. So far, efforts have been made to develop advanced strategies for producing biopeptides with specific amino acid compositions, molecular weights, and sequences through enzymatic processes.

However, defining the optimal conditions for protein hydrolysis to achieve a maximum yield of peptides with the desired activity demands significant effort. This process necessitates a systematic experimental design to identify the best conditions, which in turn lengthens the time necessary for peptide discovery.

Factors such as the type of source protein substrates, the specificity of proteolytic enzymes used for hydrolysis, and processing conditions (such as time and temperature) significantly affect the outcome [36]. The selection of an appropriate enzyme or a combination of different enzymes is one of the main critical steps [37]. The specificity of each digestive enzyme varies, and some enzyme specificities remain unknown, resulting in random peptide production with unpredictable sequences [37,38]. Moreover, processes of bioactive peptide identification involve multiple purification steps and activity determinations, which are highly time-consuming. As a result, many researchers fail to obtain BAPs due to low peptide yields after purification.

Indeed, the integration of bioinformatics has significantly enhanced the search for BAPs, providing a revolutionary approach through its robust suite of computational analytical tools. This statement is underscored by recent notable advancements and cutting-edge developments as described in the literature [37,38,39,40,41]. More specifically, machine-learning approaches in this field have significantly bolstered our ability to predict and identify BAPs with unprecedented accuracy and efficiency [42,43,44]. This up-to-date methodology is proving to be a game-changer, revolutionizing the landscape of peptide discovery and underscoring the critical role of advanced analytics in contemporary biochemical research.

This study presents a novel computational flowchart designed to identify and optimize critical enzymes involved in protein hydrolysis. This adaptable system can be applied to any protein source, efficiently extracting diverse BAPs to enhance yields and potential health benefits. The demonstrated success of this flowchart in generating diverse BAPs from tomato peels highlights its potential to unlock valuable bioactive compounds from various underutilized sources. This approach represents a significant advancement toward sustainable and efficient biopeptide production, from the same source, for multiple health and industrial applications.

## 2. Methods

### 2.1. Genome Mining and Protein Selection

The publicly accessible Affymetrix GeneChip Tomato Genome Array dataset GSE19326 (https://www.ncbi.nlm.nih.gov/geo/query/acc.cgi?acc=GSE19326) [45], which comprehensively covers gene expression data from a variety of *S. lycopersicum* plant tissues, served as the basis for our investigation. We narrowed our focus down to the peel of the red fruit cultivar, specifically Line27859. After calculating the average expression values across three replicates, we identified and selected the fifty most abundantly expressed proteins for subsequent analysis. To retrieve the sequences of these proteins, we utilized the Sol Genomics Network (https://solgenomics.net/search/expression/, accessed on 11 December 2023). Furthermore, to ensure the accuracy and reliability of our protein annotations, we cross-referenced our data with the UniProt database, filtering our list to include only those proteins that had been accurately manually annotated.

### 2.2. In Silico Digestion

The Rapid Peptide Generator (RPG, available at https://gitlab.pasteur.fr/nmaillet/rpg, accessed on 5 February 2024, [46]) was utilized to conduct in silico digestion on a collection of proteins extracted from tomato peels. To maximize the diversity of generated peptides, we opted for a sequential mode digestion strategy, simulating the combined action of two different enzymes. RPG offers a library of 40 different enzymes. We systematically explored all possible pairwise combinations of these enzymes. Importantly, we excluded the use of chemical compounds included in the RPG tool, focusing solely on enzymatic hydrolysis. In sequential mode, each protein was digested by each enzyme, one by one. All parameters were set to their default standard values. The input for this process was a FASTA file comprising the 50 most highly expressed and annotated proteins found in tomato peel. For each round of hydrolysis, a pair of enzymes was selected from the 40 different enzymes available within the RPG tool. This approach led to the simulation of all possible combinations, totaling 1560 unique enzymatic pairs. After each simulated hydrolysis, the resultant peptide sequences were captured in output files. These files were then processed to filter and retain only those peptides whose lengths were within the range of 5 to 30 amino acids (aa), ensuring the selection of peptides suitable for further bioactive potential analysis.

### 2.3. Bioactivity Peptide Prediction

Each output file, containing peptides ranging from 5 to 30 amino acids, was subjected to analysis using the Multi-label deep-learning approach to determine the multifunctionality of BAPs (MLBP, available at https://github.com/xialab-ahu/MLBP, accessed on 5 February 2024, [47]). The MLBP tool evaluates peptides for their potential to belong to multiple bioactive classes, including anticancer, antidiabetic, antihypertensive, anti-inflammatory, and antimicrobial categories. By leveraging this deep-learning approach, we aimed to identify and categorize peptides based on their predicted bioactive properties, thereby narrowing down the pool of candidate peptides for further analysis and validation. After predicting the bioactivity for each peptide in the output files, we counted the number of peptides that fell into each bioactive class. This data enabled us to identify which enzyme combinations produced the highest number of peptides for each specific bioactivity class, facilitating a targeted approach to selecting peptides with desired therapeutic properties.

### 2.4. Filtering

#### 2.4.1. Screening for Toxicity, Bitterness, and Stability

The initial filtering step involved comprehensive screening for toxicity, bitterness, and stability of the virtually generated peptide products. All peptides were subjected to multiple database searches using the peptide query in FASTA format. To predict peptide cytotoxicity, ToxinPred3 (https://webs.iiitd.edu.in/raghava/toxinpred3/prediction.php, accessed on 6 March 2024, [48]) was used, which employs a Support Vector Machine (SVM). Peptide products predicted to be toxic were excluded from further analysis. For bitterness screening, iBitter-SCM (http://camt.pythonanywhere.com/iBitter-SCM/, accessed on 6 March 2024, [49]) was utilized. Peptides scoring above a threshold of 333 were predicted to have a bitter taste and were therefore excluded from subsequent analyses. The stability of peptides in the bloodstream was assessed using PLifePred (https://webs.iiitd.edu.in/raghava/plifepred/, accessed on 6 March 2024, [50]), and peptides with a predicted half-life exceeding 800 s were included from the study [51]. Additionally, the HLP (http://crdd.osdd.net/raghava/hlp/interactive.htm/, accessed on 6 March 2024, [52]) tool was used to evaluate peptide stability in the intestinal environment, retaining only peptides predicted to be stable under these conditions. The 1-s threshold was chosen based on the HLP webserver’s classification system, which defines peptides as highly stable if they have a half-life greater than 1.0 s.

#### 2.4.2. Bioactivity Evaluation

Following the initial screening, the remaining non-toxic, non-bitter, and stable peptides were subjected to further evaluation. In particular, peptides were screened against specific bioactivity platforms to confirm BAPs selected within the antihypertensive, antidiabetic, anti-inflammatory, anticancer, and antimicrobial classes. The web servers used for bioactivity prediction included in this study (accessed on 8 March 2024) are reported in Table 1. This dual-step filtering process ensured that only the most promising peptides, in terms of safety, stability, and potential bioactivity, were selected for further analysis. Webservers used in this study (accessed on 8 March 2024) are reported in Table 1.

### 2.5. Peptides Structure Prediction

The three-dimensional structures of the peptides selected from the previous phases were predicted using PepFold4 (https://bioserv.rpbs.univ-paris-diderot.fr/services/PEP-FOLD4/, accessed on 8 March 2024). PepFold4 is a modeling server that predicts the structure of short peptide chains based on their low-energy conformations. The peptide sequences were submitted to the server, and the three-dimensional structure predictions were carried out. For each prediction, the structure of the representative pose from the most representative cluster was downloaded. These structural models were then used for further analyses.

### 2.6. Molecular Docking

Following the prediction of the three-dimensional structures of the selected peptides for each class, potential protein targets associated with the bioactivity of the peptides were then identified. For the ACPs, the target protein selected was human aminopeptidase N (PDB: 4FYQ). The AHPs targeted the Human Angiotensin Converting Enzyme (PDB: 1O86), the ACPs targeted Human DPP-IV (PDB: 2ONC), and the AIPs targeted Human INOSOX (PDB: 3E7G). For AMPs, different target proteins of the principal multidrug-resistant strains were selected, as follows: penicillin-binding protein 1a (PDB: 3UDF) of *Acinetobacter baumanii*; oxygen-intensive NADPH nitroreductase (PDB: 3QDL) of *Helicobacter pylori*; UDP-N-acetylmuramoyl-L-alanyl-D-glutamate-L-lysine ligase (PDB: 4C12) of *Staphylococcus aureus*; Streptomycin 3″-adenylyltransferase (PDB: 6FZB) of *Salmonella enterica*; and Metallo-beta-lactamase type 2 (PDB: 6EW3) of *Pseudomonas aeruginosa*. Subsequently, a molecular-docking analysis was conducted using the software FRODOCK (v3.12) (http://frodock.chaconlab.org, accessed on 11 March 2024). This software was utilized to predict the binding interactions between the peptides and their respective protein targets. FRODOCK (v3.12) generates the top five binding poses for each protein–peptide interaction. These poses were then analyzed to determine the most favorable binding configurations, which could provide insights into the potential efficacy and mechanism of action of each BAP.

### 2.7. Energy Calculation and Interaction Analysis

#### 2.7.1. Molecular Dynamics for Re-Scoring and Energy Calculation

Each binding pose provided by FRODOCK was utilized to conduct molecular dynamics analysis using GROMACS software (https://www.gromacs.org, v.2023.2, accessed on 11 March 2024) to perform pose re-scoring. Each system underwent a two-stage minimization process. Initially, side chains were minimized for 5000 steps using the steepest descent method, while the backbone was restrained. This was followed by a similar steepest descent minimization of the entire system for another 5000 steps. The equilibration of each complex was performed in two stages. The first stage involved simulating the system for 500 ps under the NVT ensemble at a constant temperature of 310 K using the V-rescale thermostat. Here, a 1000 kj/mol force was applied to restrain the position of heavy atoms to facilitate solvent equilibration. The second stage aimed to improve side-chain solvation through ‘soft equilibration’, applying a 100 kj/mol force on heavy atoms. This stage lasted another 500 ps, but under NPT conditions, with pressure maintained at 1.0 bar using the Parrinello–Rahman barostat. Finally, 1 ns of unrestrained production was performed.

This approach allowed the simulation of the dynamic behavior of peptide–protein interactions in an aqueous environment. Subsequently, the gmx_MMPBSA (Molecular Mechanics Poisson-Boltzmann Surface Area) tool was used to perform end-state free energy calculations [60]. The selected binding poses were filtered based on their binding energy, retaining only those with an energy equal to or less than −30 kcal/mol.

#### 2.7.2. Interaction Analysis

From the selected poses, key interactions between peptides and amino acids in the active site of the target enzyme were identified. This analysis was conducted using LigPlot+ v.2.2 (https://www.ebi.ac.uk/thornton-srv/software/LigPlus/, accessed on 3 April 2024, [61]), which provides detailed visualization of non-covalent interactions. Specifically, hydrogen bonds that could influence the stability and affinity of the peptide–protein complex were identified and evaluated.

## 3. Results

### 3.1. Workflow for the Identification and Evaluation of Bioactive Peptides

**Figure 1:**
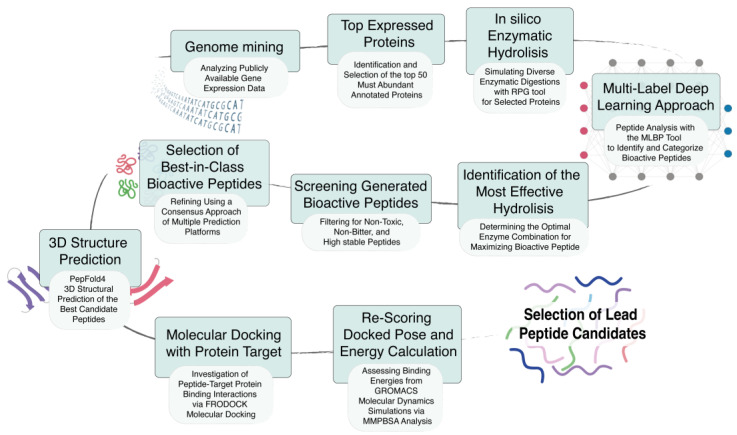
Comprehensive workflow for the identification and evaluation of BAPs from RNA sequence data to the final selection of the best candidate peptides. Genome mining: This initial step involves analyzing publicly available gene expression data to identify potential sources of BAPs; Top Expressed Proteins: Identification and selection of the top 50 most-abundant annotated proteins based on gene expression data. These proteins serve as the starting material for peptide generation; In silico Enzymatic Hydrolysis: Simulation of diverse enzymatic digestions using the RPG tool to cleave the selected proteins into smaller peptides; Multi-Label Deep-Learning Approach: Application of the Multi-Label Deep-Learning Tool (MLBP) to analyze the generated peptides, identifying and categorizing them based on their potential bioactivities; Identification of the Most Effective Hydrolysis: Determining the optimal enzyme combinations that maximize the production of BAPs; Screening Generated Bioactive Peptides: Filtering the generated peptides to select those that are non-toxic, non-bitter, and highly stable, ensuring the identification of safe and effective candidates; Selection of Best-in-Class Bioactive Peptides: Refining the selection of peptides using a consensus approach involving multiple prediction platforms to identify the most promising BAPs; 3D Structure Prediction: Using PepFold4 to predict the 3D structures of the best candidate peptides, providing insights into their spatial configuration and potential interactions. Molecular Docking with Protein Target: Investigation of peptide-target protein-binding interactions via FRODOCK molecular docking, which helps to assess the affinity and specificity of peptides for their target proteins. Re-Scoring Docked Pose and Energy Calculation: Assessing binding energies using GROMACS molecular dynamics simulations and MMPBSA analysis to evaluate the stability and strength of peptide–protein interactions; Selection of Lead Peptide Candidates: Based on the preceding analyses, the most promising peptides are selected as lead candidates for further experimental validation and potential therapeutic development.

The workflow, visually represented in Figure 1, provides a detailed elucidation of the step-by-step computational methodology employed in this study. This comprehensive approach facilitates the identification and thorough characterization of BAPs, specifically those derived from the most abundant proteins found within tomato peels. Each stage of the workflow leverages computational tools and databases to ensure a robust and efficient analysis.

The workflow starts with genome mining and the subsequent pre-processing of data (Figure 1). This crucial step allows for the comprehensive identification and classification of the proteins inherently present within tomato peels. Leveraging the power of bioinformatics, we have analyzed publicly available gene expression data like GSE19326 [45], which encompasses gene expression data from various tissues of *S. lycopersicum* plants. Our analysis has focused specifically on the cultivar Line27859. This emphasis stems from high by-product yield. These by-products, including peels, offer significant economic potential as they are rich in valuable bioactive compounds.

After initial protein profiling, we focused on the 50 most abundant proteins in the tomato peel proteome (Figure 1; Supplementary Data File). This selection was based on their high expression levels, which are likely to be the most relevant substrates for generating BAPs. These peptides potentially include antioxidants, antimicrobials, and antihypertensives, which are significant for their health-promoting properties

Following this initial protein profiling, we narrowed our focus to the 50 most abundant proteins identified in the tomato peel proteome (Figure 1; Supplementary Data File). This selection was based on their high expression levels, which are likely to be the most relevant substrates for generating BAPs. These peptides potentially include antioxidants, antimicrobials, and antihypertensives, which are significant for their health-promoting properties.

The selected proteins are then subjected to in silico enzymatic hydrolysis using the Rapid Peptide Generator (RPG) tool [46] (Figure 1). This computational simulation replicates the process of enzymatic protein breakdown, utilizing a diverse set of 40 hydrolytic enzymes to generate an array of smaller peptide fragments. By employing this approach, we create a comprehensive roster of potential peptides that could be derived from the most abundant tomato peel proteins (Supplementary Data File).

The resulting pool of simulated peptides, comprising a different range of lengths, underwent an initial filtration process to retain only those with lengths between 5 and 30 amino acid residues. This refined set of peptides was then subjected to in silico screening using a state-of-the-art Multi-Label Deep-Learning approach (MLBP) specifically designed for predicting peptide bioactivity (Figure 1). This powerful predictive model enables us to identify peptides with promising potential for exhibiting five key therapeutic activities with anticancer (ACP), antidiabetic (ADP), antihypertensive (AHP), anti-inflammatory (AIP), and antimicrobial (AMP) effects. By employing this advanced computational method, we can efficiently evaluate a large number of peptide candidates for their potential bioactive properties, streamlining the process of identifying the most promising peptides for further investigation (Supplementary Data File).

The computational workflow proceeds with the identification of key enzymes involved in protein hydrolysis through multi-enzyme screening (Figure 1). This crucial step allows us to determine the most effective enzyme combinations that yield the highest abundance of BAPs across different therapeutic classes (anticancer, antidiabetic, antihypertensive, anti-inflammatory, and antimicrobial). By optimizing the enzyme selection, we can maximize the potential for generating peptides with variegated bioactive properties from the tomato peel proteins (Supplementary Data File).

To ensure the safety and therapeutic viability of our candidate peptides, we implemented a rigorous filtering step (Figure 1). This critical stage employs a suite of prediction tools—ToxinPred3, iBitter-SCM, HLP, and PLifePred. This comprehensive in silico screening process effectively eliminates peptides predicted to exhibit toxicity, bitterness, or instability, focusing solely on those demonstrating high intestinal and plasma stability—crucial properties for oral bioavailability and therapeutic efficacy. By applying these advanced computational methods, we refined our selection to peptides that not only show promising bioactive potential but also meet essential criteria for safety and stability in physiological environments (Supplementary Data File).

The peptides that successfully pass the initial safety and stability filters undergo a more comprehensive and targeted evaluation. This phase employs specialized bioactivity prediction platforms designed to confirm and refine the predicted activities within the five key therapeutic classes—anticancer (ACP), antidiabetic (ADP), antihypertensive (AHP), anti-inflammatory (AIP), and antimicrobial (AMP). This confirmatory analysis harnesses the predictive power of multiple tools, namely ACPred, MLACP 2.0, and ACPPfel for ACP; iDPPIV-SCM for ADP; AHTpin for AHP; AIPpred and PreTP-Stack for AIP; and CAMPR3 for AMP. This multi-tiered filtering approach ensures a high level of confidence in the predicted bioactivity profiles of the candidate peptides (Figure 1; Supplementary Data File). By utilizing these specialized tools, we can more accurately identify peptides with the greatest potential for exhibiting the desired therapeutic properties.

Following bioactivity prediction and filtering, we modeled the three-dimensional structures of the promising peptides using PEP-FOLD4 [62]. These structural models then served as the foundation for a detailed molecular docking analysis conducted with FRODOCK (v3.12) (Figure 1). This analysis allows us to investigate, at the molecular level, the intricate binding interactions between each peptide and its respective target protein.

We calculated the free binding energy using the Molecular Mechanics Poisson–Boltzmann Surface Area (MM-PBSA) method to refine our understanding of these interactions further. This approach is widely used for analyzing protein–protein, protein–nucleic acid, and protein–ligand interactions [63,64,65,66]. This approach provides a quantitative measure of binding affinity and was used to re-rank the top five models predicted by FRODOCK (Figure 1). The re-ranking approach in protein–protein interaction is considered more suitable [67] as most docking tools base their scoring strategies on scoring functions incorporating only a few energy terms. By combining structural modeling, molecular docking, and binding energy calculations, we gain comprehensive insights into the potential effectiveness of these peptides in their targeted therapeutic roles.

This comprehensive, multi-step workflow culminates in the identification of highly promising peptide sequences derived from tomato peel, predicted to exhibit both high bioactivity and therapeutic potential.

### 3.2. Simulated Enzymatic Hydrolysis

**Figure 2:**
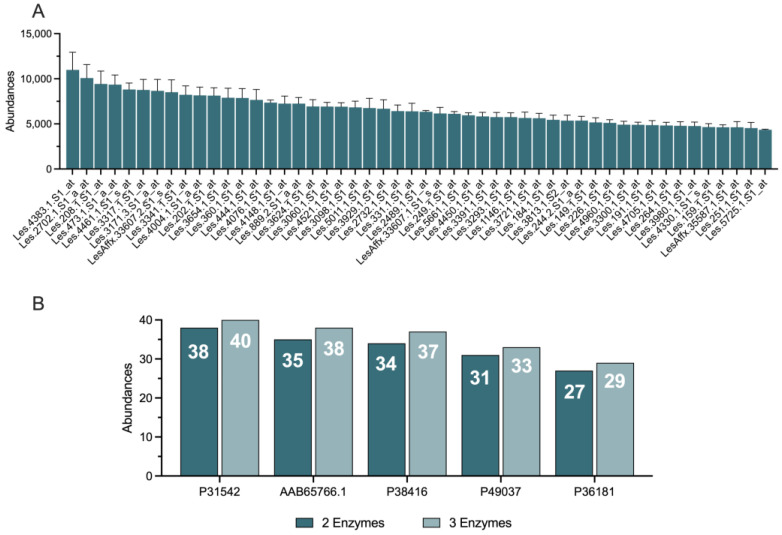
Protein analysis of *S. lycopersicum* Line27859 peel. (**A**) The panel shows the abundances of the top 50 expressed and annotated proteins in the peel of *Solanum lycopersicum* Line27859. Each bar corresponds to a specific protein, identified by its annotation code, with the height of the bar indicating the abundance level of the protein. The error bars represent the standard deviation, reflecting the variability in the data. (**B**) The panel illustrates a comparison of the number of peptides generated from the top five most abundant proteins using combinations of two and three enzymes. Each bar represents the number of peptides produced, with dark blue bars indicating the use of two enzymes and light blue bars indicating the use of three enzymes. The annotation codes of the top five proteins are listed along the x-axis (e.g., P31542, AAB65766.1), and the number of peptides generated is indicated at the top of each bar.

We performed in silico enzymatic hydrolysis on the 50 most abundant proteins identified in *S. lycopersicum* (Figure 2A). This simulation utilized the RapidPeptideGenerator tool [46], which provided a comprehensive repertoire of 40 different enzymes. To maximize the diversity of potential peptides generated, we employed a sequential digestion mode, simulating the combined action of two distinct enzymes. This approach yielded 1560 unique enzyme pairs, resulting in the generation of 11,000 total protein fragments. Notably, this extensive peptide library was achieved using the optimal enzyme combination identified through the simulation. To prioritize peptides with enhanced novelty and specificity to *S. lycopersicum* while ensuring their potential bioavailability, we applied a length-based filter. Only peptides composed of 5 to 30 amino acid residues were retained for further analysis. This crucial selection step significantly reduced the candidate pool to approximately 1200 unique peptide sequences, all generated using the most effective enzyme combination. Figure 2B showcases the resulting peptide profiles for the top five proteins, selected based on their high yield of peptides. These proteins, identified by their unique accession numbers (UniProt ID: P31542, AAB65766.1, P38416, P49037, and P36181), represent promising targets for bioactive peptide production.

To further explore the potential of generating an even more diverse peptide library, we investigated the impact of incorporating a third digestion enzyme. This expanded analysis explored 60,840 possible enzyme combinations, focusing on the top five proteins. However, as illustrated in Figure 2B, the addition of a third digestion step only marginally increased the number of generated peptides. This finding suggests that the two-step enzymatic hydrolysis, already capable of yielding a substantial and assorted peptide library, strikes an optimal balance between maximizing peptide diversity and minimizing computational resources. Therefore, we concluded that the two-step hydrolysis strategy is sufficient for further investigation into the bioactive potential of these tomato-derived peptides.

### 3.3. Prediction of Solanum lycopersicum Bio-Active Peptides (BAPs)

**Figure 3:**
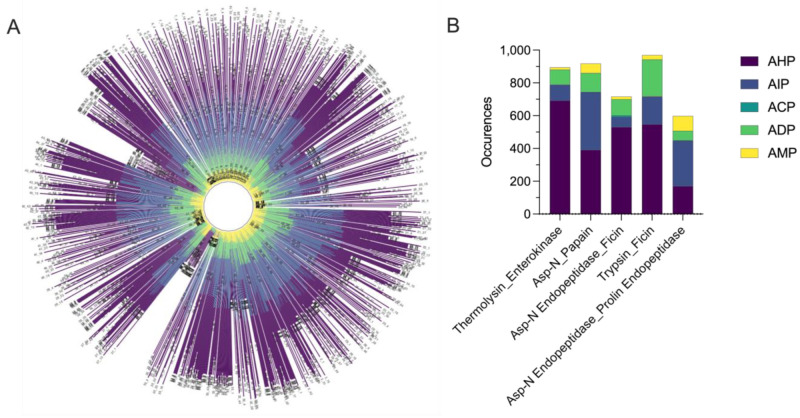
Analysis of Peptide Generation by Enzyme Combinations. (**A**) Peptide class distribution of peptides generated by each sequential dual-enzyme combination. (**B**) Peptide yield optimization shows the total number of peptides generated within each class, highlighting the enzyme combination that yielded the highest number of peptides for each specific class.

The digested peptides from the combination of two enzymes were evaluated using MLBP (Multi-label deep-learning approach for determining the multi-functionalities of Bioactive Peptides) [47] to identify peptides with potential bioactivities (Figure 3A).

From the candidate pool of 1560 combined enzymes, five were shortlisted based on their maximum numbers of predicted bioactivities in various functions, including anticancer (ACP), antidiabetic (ADP), antihypertensive (AHP), anti-inflammatory (AIP), and antimicrobial (AMP) functions (Figure 3B). This analysis serves to identify the optimal enzyme combinations for maximizing peptide diversity and tailoring peptide properties for specific downstream applications. Results showed that each class of BAPs is enriched in the following specific enzyme combinations: ACP: Asp-N Endopeptidase_Ficin (*n* = 10); ADP: Trypsin_Ficin (*n* = 224); AHP: Thermolysin_Enterokinase (*n* = 692); AIP: Asp-N_Papain (*n* = 351); and AMP: Asp-N Endopeptidase_Prolin Endopeptidase (*n* = 91) (Figure 3B, Table 2).

To ensure the selection of peptides with desirable bioactivity and safety profiles, a rigorous multi-step filtering process was implemented. Initially, the peptide candidates derived from *S. lycopersicum* were screened using a suite of computational tools—ToxinPred3, iBitterSCM, HLP, and PLifePred. This analysis aimed to eliminate peptides predicted to exhibit toxicity or bitterness while prioritizing those with high stability in both intestinal and blood environments (Table 2). Specifically, the HLP and PLifePred analyses revealed the following subset of peptides demonstrating exceptional stability: 4 anticancer peptides (ACP), 49 antioxidant peptides (ADP), 220 antihypertensive peptides (AHP), 66 anti-inflammatory peptides (AIP), and 13 antimicrobial peptides (AMP). These peptides exhibited an intestinal half-life exceeding 1.0 s and a predicted plasma half-life of 800 s or greater, indicating robust stability within the human body [51].

Only the unique peptides successfully passing all selection criteria—non-toxic, non-bitter, and possessing high intestinal and blood stability—were retained for subsequent in-depth analysis. This stringent selection process ensures a focused investigation of peptides with a higher likelihood of exhibiting bioactivity and safety in potential applications.

### 3.4. Prospect Solanum lycopersicum Bioactive Peptides

The pool of promising BAP candidates identified in Table 2 underwent further stringent selection based on predicted bioactivities assessed using multiple in silico platforms. This comprehensive approach aimed to identify high-potential BAPs exhibiting robust and consistent evidence of bioactivity. Peptides were considered strong candidates if they met two key criteria: (i) High predictive model scores; candidates were required to achieve scores surpassing the established thresholds for each specific bioactivity prediction model employed. This ensured that only peptides predicted with high confidence to exhibit the desired bioactivity were considered. (ii) Consistent binding site interactions; to further validate the bioactivity predictions, the shortlisted peptides were subjected to molecular docking simulations. Candidates demonstrating consistent and favorable interactions with known binding sites associated with the target bioactivity were deemed high-potential BAPs.

This dual-pronged approach, leveraging both predictive models and binding site analysis, ensured the selection of BAPs with a higher likelihood of exhibiting genuine bioactivity, warranting further experimental validation.

#### 3.4.1. Anticancer Peptides (ACPs)

To identify the most promising ACPs from the *S. lycopersicum*-derived peptide, a robust selection strategy was employed leveraging three state-of-the-art ACP prediction tools, namely ACPred, MLACP 2.0, and ACPPfel. These servers represent the most recent and performance-optimized tools, each employing distinct algorithms and datasets for enhanced prediction accuracy. Peptides identified as positive hits by all three platforms were designated top-tier ACP candidates, signifying high confidence in their predicted activity. This stringent consensus-based approach minimized the likelihood of false positives and prioritized peptides with robust evidence from multiple prediction models. Remarkably, this rigorous screening process yielded two promising ACPs. The decapeptide MWKLPMFGCT emerged as a strong candidate, exhibiting consistent optimistic predictions across all three platforms. The pentapeptide WKLPM, representing a fragment of the more extensive MWKLPMFGCT sequence, was also identified as a potential ACP.

To further investigate the potential mechanism of action of these ACP candidates, molecular docking simulations were performed using FRODOCK [68]. The simulations focused on the interactions of both peptides with the crystal structure of a known anticancer target protein, Aminopeptidase N (PDB ID: 4FYQ), identified in a previous study [69]. The docking results, summarized in Table 3, revealed that MWKLPMFGCT and WKLPM exhibit favorable binding interactions with key catalytic residues of the target protein (Supplementary Figure S1). This finding provides compelling evidence supporting the potential anticancer activity of these peptides and warrants further experimental validation.

#### 3.4.2. Antidiabetic Peptides (ADPs)

High-potential ADPs were identified based on their scores meeting or going beyond the established threshold of the predictive model, along with consistent interaction observed at known binding sites associated with its target bioactivity. Specifically, peptides were considered ADPs if their iDPPIV-SCM scores exceeded 300 and based on their predicted ability to inhibit dipeptidyl peptidase IV (DPP-IV) (PDB ID: 2ONC), a well-established therapeutic target for managing type 2 diabetes (Table 4). From the initial pool of peptides, those predicted to be ADPs by the iDPPIV-SCM model were selected. This initial screening yielded 20 promising candidate peptides from the initial 46 peptides. To further refine the selection and ensure potential efficacy, these peptides underwent molecular docking simulations to identify peptides that could effectively bind to DPP-IV and inhibit its activity. Peptides exhibiting binding sites with DPP-IV were considered high-potential ADPs. Of the 20 initial candidates, 10 peptides demonstrated strong binding affinities to DPP-IV, exhibiting MMGBSA values less than −30 kcal/mol (Table 4). Notably, two peptides, QEFAHDFQAY and HWLNTHAVIE, exhibited interactions specifically with the catalytic residues of DPP-IV (Supplementary Figure S2). This finding suggests their potential to interfere with the enzyme’s catalytic activity directly, further strengthening their potential as potent DPP-IV inhibitors. Identifying these high-potential ADPs, particularly those interacting with DPP-IV’s catalytic residues, presents a promising avenue for developing novel therapeutics for type 2 diabetes.

#### 3.4.3. Antihypertensive Peptides (AHPs)

Promising AHPs from *S. lycopersicum* were selected by integrating predictive model scores and molecular docking simulations. This approach aimed to identify peptides exhibiting high predicted activity and favorable interactions with known antihypertensive targets. Specifically, nine AHP candidates were chosen based on (i) high AHTpin scores—all selected peptides exhibited AHTpin scores exceeding 1.0, indicating a high probability of antihypertensive activity based on the predictive model; and (ii) predicted binding to Angiotensin Converting Enzyme (ACE) (PDB ID: 1O86)—molecular docking simulations using FRODOCK and GROMACS re-scoring were employed to assess the binding affinity and interactions of each peptide with the active site residues of human ACE, a key enzyme involved in blood pressure regulation. This analysis ensured the selected peptides exhibited favorable binding characteristics to a validated antihypertensive target.

Table 5 presents a detailed analysis of these nine AHP candidates, encompassing their sequences, AHTpin scores, predicted binding affinities to ACE, and key molecular interactions identified through the docking and molecular dynamics simulations (Supplementary Figure S3). This comprehensive in silico characterization provides a strong foundation for prioritizing these peptides for further experimental validation of their antihypertensive potential.

#### 3.4.4. Anti-Inflammatory Peptides (AIPs)

Three independent computational methods, MLBP, PreTP-Stack, and AIPpred, were employed to predict potential AIPs. This step aimed to refine the selection based on their predicted anti-inflammatory properties. Subsequently, the molecular docking software was utilized to simulate and analyze the interactions between the selected AIPs and the target protein inducible nitric oxide synthase (iNOS) (PDB ID: 3E7G). Of the 48 initial candidates, 8 peptides exhibited significant binding affinities to the target protein, with MMGBSA values less than −30 kcal/mol (Table 6). Notably, four of these peptides—CGYSMNSIEGAAVSTIHITPE, DETPELMPLSHVLATKLGAR, PTKGSSVAIFGLGAVGLAAAEGAR, and NQKNLHKRYAYQIVLQTREMLR—demonstrated interactions specifically within the protein’s dimerization region (Supplementary Figure S4). This observation suggests their potential to disrupt protein dimerization, a mechanism often associated with modulating inflammatory responses.

#### 3.4.5. Antimicrobial Peptides (AMPs)

Promising AMPs from *S. lycopersicum* were selected by integrating predictive model scores of MLBP and CAMPR3, as well as molecular docking simulations. Research on AMPs focuses on exploring their structural and functional diversity, mechanisms of action, and therapeutic potential against various bacterial infections, including multidrug-resistant strains. To this aim, we selected some target proteins of the principal multidrug-resistant strains, such as penicillin-binding protein 1a (PDB: 3UDF) of *Acinetobacter baumanii*, oxygen-intensive NADPH nitroreductase (PDB: 3QDL) of *Helicobacter pylori*, UDP-N-acetylmuramoyl-L-alanyl-D-glutamate-L-lysine ligase (PDB: 4C12) of *Staphylococcus aureus*, Streptomycin 3″-adenylyltransferase (PDB: 6FZB) of *Salmonella enterica*, and Metallo-beta-lactamase type 2 (PDB: 6EW3) of *Pseudomonas aeruginosa*.

Table 7 presents a detailed analysis of these AMP candidates, encompassing their sequences, predicted binding affinities to the target protein, and key molecular interactions identified through the docking and molecular dynamics simulations (Supplementary Figures S5–S9).

## 4. Discussion

This study presents a robust computational workflow to uncover and characterize BAPs hidden within tomato peel proteins. By harnessing the power of proteomic analysis, in silico enzymatic hydrolysis, and multi-label deep learning, we identified peptides with promising therapeutic potential across various domains, including anticancer, antidiabetic, antihypertensive, anti-inflammatory, and antimicrobial activities.

Conventional methods for producing BAPs from food by-products include enzymatic digestion, fermentation (microbial digestion), or combining both techniques [70,71,72,73,74,75,76]. These methods typically involve selecting a protein source, hydrolyzing it with a chosen enzyme, purifying and identifying the resulting peptides, and evaluating their biological activities [77]. Moreover, pretreatments (e.g., high-pressure, ultrasound, and microwave treatment) occasionally enhance peptide release and reduce interferences during biological activity analyses [78,79]. These procedures are time-consuming and resource-intensive compared to discovering new BAPs using in silico processes [80]. Computational approaches enable the efficient selection of appropriate enzymes for protein sources and eliminate the guesswork associated with conventional methods. They also predict possible biological activity and determine action mechanisms through molecular docking [81,82,83].

Our process began with mining proteomic data from tomato peels, a readily available by-product brimming with bioactive compounds thanks to their high protein content. Utilizing proteomic and gene expression profiles, specifically from the tomato cultivar Line27859 (GSE19326), we meticulously selected the 50 most abundant proteins as our starting point, ensuring the relevance and potential of our chosen substrates. Mimicking nature’s intricate processes, we employed the Rapid Peptide Generator tool to simulate in silico enzymatic hydrolysis. This approach generated a diverse library of potential peptides by sequentially applying two distinct enzymes chosen from a pool of 1560 possible pairs of 40 enzymes. Filtering this library for peptides with lengths between 5 and 30 amino acids ensured their bioavailability and stability. The refined peptide pool then underwent rigorous screening using a multi-label deep-learning approach (MLBP), which is capable of simultaneously predicting multiple bioactivities. This step allowed us to pinpoint peptides with high probabilities of exhibiting therapeutic benefits. To further refine our selection, we employed tools like ToxinPred3, iBitter-SCM, HLP, and PLifePred to assess safety and stability, retaining only non-toxic and non-bitter peptides with high intestinal and plasma stability. A similar process enabled the screening of toxicity, bitterness, and stability in the intestine and blood of BAPs derived from oyster proteins, which were virtually digested under simulated gastrointestinal conditions [84].

This selection process, while enhancing the likelihood of identifying safe and potential BAPs, could exclude some BAPs that do not meet these stringent criteria. This limitation might impact the discovery of peptides with unique or potent bioactivities but with less favorable stability or taste profiles. Additionally, the advantages of computational predictions could be inherently limited by the potential for false positives and negatives. For instance, the bioactivity of a peptide can be overestimated if the model has not been trained on a sufficiently diverse dataset, or it can miss potential bioactivities due to the limitations in understanding the complex mechanisms of interaction between peptides and biological targets. Therefore, more studies are necessary to explore alternative selection criteria or parallel screening approaches to mitigate these potential biases.

Our workflow yielded a diverse set of promising candidates, each with specific bioactivity, all of which were subjected to in-depth characterization using a suite of computational tools to validate predicted activities and analyze molecular interactions. For anticancer peptides, diverse prediction models were used to predict activity followed by molecular docking simulations to assess interactions with known anticancer targets. Peptides like MWKLPMFGCT and its fragment WKLPM emerged as promising candidates, exhibiting favorable binding interactions and low free binding energies. Similarly, antidiabetic peptides were evaluated based on their predicted ability to inhibit DPP-IV, an essential target protein in diabetes management. Peptides like QEFAHDFQAY and HWLNTHAVIE demonstrated strong binding affinities to DPP-IV interacting with key residues, suggesting their potential as effective inhibitors. Our analysis also identified nine antihypertensive peptide candidates based on high scores and favorable interactions with ACE. Molecular docking simulations confirmed their binding characteristics, highlighting their potential as antihypertensive agents. For instance, the peptide VEMQDVKYP exhibited significant binding affinity and interactions with key residues, underscoring its therapeutic potential. Out of the 48 initial AIP candidates, four of these peptides—CGYSMNSIEGAAVSTIHITPE, DETPELMPLSHVLATKLGAR, PTKGSSVAIFGLGAVGLAAAEGAR, and NQKNLHKRYAYQIVLQTREMLR—exhibited significant binding affinities to the target protein and demonstrated interactions specifically within the protein’s dimerization region. This observation suggests their potential to hinder proper dimer formation, impairing enzymatic function. When screening antimicrobial peptides against target proteins of the leading multi-drug-resistant bacterial strains, it was found that three particular peptides—DAGASKTYPQQAGTIRKGGHIVIKNRP, DGPNASYITPAAL, and DFLIGNTSTGYCAGGCAAIV—have the ability to bind to crucial amino acid residues. This binding occurs consistently across all the target proteins tested from various bacterial species.

The discovery of these BAPs in tomato peels holds high promise for developing functional foods and nutraceuticals [85,86]. By incorporating these peptides into food products, we can enhance their nutritional profile and deliver health benefits beyond essential nutrition [87,88,89].

This comprehensive, multi-step computational workflow culminates in successfully identifying the most promising peptide sequences derived from tomato peel proteins. These peptides are predicted to exhibit high bioactivity and significant therapeutic potential across multiple health-related domains. By integrating advanced proteomics, machine learning, molecular modeling, and docking simulations, we have systematically narrowed down a vast pool of potential peptides to select candidates. These peptides demonstrate predicted efficacy in various therapeutic applications and meet crucial criteria for safety, stability, and target interaction. However, effectiveness in terms of the efficacy and safety of the predicted bioactivities of these peptides requires their validation through in-vitro and, where necessary, in vivo experiments.

These biological validation studies are also necessary to overcome the issue related to the frequency of peptide release from the protein structure by a single protease or protease complex. This aspect was not explicitly modeled in our study as, due to different protein conformations, some sites are inaccessible for hydrolysis, while hydrolysis on the linear protein sequence shows a much better effect.

Finally, our study illuminates the huge potential of tomato peels as a rich source of bioactive peptides, further supporting the advantages of developing health-enhancing foods and dietary supplements and valorizing tomato peels aligns perfectly with sustainable practices by reducing food waste and contributing to a circular economy.

## Figures and Tables

**Table 1:** Prediction tool servers used for filtering the BAPs.

Bioactivity	Tool	Site	Ref.
ACP	ACPred	http://codes.bio/acpred/ (accessed on 8 March 2024)	[53]
	MLACP2	https://balalab-skku.org/mlacp2/ (accessed on 8 March 2024)	[54]
	ACPPfel	http://lmylab.online:5001/ (accessed on 8 March 2024)	[55]
AHP	AHTpin	http://crdd.osdd.net/raghava/ahtpin (accessed on 8 March 2024)	[56]
ADP	iDPPIV-SCM	http://camt.pythonanywhere.com/iDPPIV-SCM (accessed on 8 March 2024)	[57]
AIP	AIPpred	http://211.239.150.230/AIPpred/AIPpredMethod.html/ (accessed on 8 March 2024)	[58]
AMP	CAMP_R3_	http://www.camp3.bicnirrh.res.in/ (accessed on 8 March 2024)	[59]

**Table 2:** Peptide candidate predicted using MLBP (“Bioactive Class”) and pre-screened for toxicity (“Toxic” column), bitterness, and stability in both intestinal and blood environments (“Non-Toxic, Non-Bitter, High Stable” column).

Bioactive Class	Total	Toxic	Non-Toxic Non-Bitter High Stable
ACP	10	6	4
ADP	187	36	49
AHP	692	144	220
AIP	351	9	66
AMP	91	15	13

**Table 3:** Top *S. lycopersicum* anticancer peptides candidate with prediction score results and interacting residues for the target protein.

Peptide Sequence	Accession Number	Protein Name	Residues Number	MMGPBSA (kcal/mol ± SD)	Target Binding Residues	Peptide Binding Residues
MWKLPMFGCT	P05349	Ribulose bisphosphate carboxylase small subunit, chloroplastic 4	10	−52.98 ± 2.58	G352 *, A353 *, R381, R442	K3, P5, C9
WKLPM	P05349	Ribulose bisphosphate carboxylase small subunit, chloroplastic 4	5	−45.91 ± 1.77	Q213, E355 *, E389 *, Y477 *	W1, K2, L3

* Residues of the active site. The main catalytic residues are G352-N356, H388, E389, H392, E411, and Y477 [UniProt: P15144].

**Table 4:** Top *S. lycopersicum* antidiabetic peptides candidate with prediction score results and interacting residues for the target protein.

Peptide Sequence	Accession Number	Protein Name	Residues Number	MMGPBSA (kcal/mol ± SD)	Target Binding Residues	Peptide Binding Residues
DLLNIFE	Q84K11	Sine/threonine-Ptein phosphatase-5	7	−41.36 ± 1.47	R122, E202, E203, S206, Y544, R666	D1, L2, N4, I5
LMAALNLVG	P37222	NADP-dependent malic enzyme, chloroplastic	9	−38.08± 1.52	E202, E203, G629	L1, N6
HWLNTHAVIE	P38416	Linoleate 9S-lipoxygenase B	10	−36.91 ± 3.16	E202, E203, Y544, R666, N707	H1, N4, T5, E10
TFAFQAE	P36181	Heat shock cognate Ptein 80	7	−36.46 ± 5.99	E202, E203, Y544, S624, S627, Y659, D660	T1, Q5, E7
QFVTFMK	Q6DUX3	Translationally controlled tumor Protein homolog	7	−35.22 ± 6.75	Y659, N706, N707, D736	M6, K7
QEFAHDFQAY	P05117	Polygalacturonase-2	10	−33.71 ± 4.20	R557, K551, S627 *	F7, Q8, A9
LPHPDGDQFG	P38416	Linoleate 9S-lipoxygenase B	10	−33.24 ± 1.29	R122, Y544, P547, K551, Y626, Y659	L1, P2, H3, D5
GWAPQVLLLS	K4D422	UDP-Glycosyltransferase 73C4	10	−31.93 ± 2.32	R122, E202, S206	G1, Q5
LAQNNVMFE	NP_001294934	Fructose-bisphosphate aldolase	9	−30.91 ± 1.43	R122, E202, E203, C548, Y582	L1, Q3, N5, E9
EMIWDLLVS	Q40140	Dartic Ptease	9	−30.7 ± 4.15	K119, R557, N559, D736, E738	E1, I3, L7, V8

* Residues of the active site. The main catalytic residues are S627, D705, and H737 [UniProt: P27487].

**Table 5:** Top *S. lycopersicum* antihypertensive peptides candidate with prediction score results and interacting residues for the target protein.

Peptide Sequence	Accession Number	Protein Name	Residues Number	MMGPBSA (kcal/mol ± SD)	Target Binding Residues	Peptide Binding Residues
MEMGESP	Q40158	Metallothionein-like Ptein type 2 B	7	−60.77 ± 6.10	Q886, E989, H992, E1016 *, K1112, H1114, Y1121	M1, E2, E5, S6
VEMQDVKYP	NP_001234021	Polygalacturonase-2 precursor	9	−55.91 ± 3.41	H992, Y999, R1007, Q1008, E1016 *, R1123	V1, E2, M3, Q4, K7
MEEVDVAPPQK	P28032	Alcohol dehydrogenase 2	11	−54.94 ± 6.25	Y667, N671, N690, R729, Y740, E989 *, Y999, E1008, E1016 *, R1123,	E2, E3, D4, P9, Q10, K11
ANQPLPDDDDEA	P38546	GTP-binding nuclear Ptein Ran1	12	−50.70 ± 10.09	N671, R719, K723, D726, R729, Y818, Y965, R1123	A1, N2, Q3, P4, D7, D8, D9
VIPKENN	P30264	Catalase isozyme 1	7	−50.18 ± 7.84	E767, Q886, D982, V984, V985, K1055, K1112, Y1124	V1, K4, E5, N6, N7
IDWKETPEPH	P30221	Class I heat shock Protein	10	−44.58 ± 6.55	D726, R729, Y829 *, D963, Y965 E1008, S1118 *, R1123	I1, D2, W3, K4, E5, T6, E8, H10
FEKGTHIPP	P37222	NADP-dependent malic enzyme	9	−41.23 ± 1.85	N671, A959, D963, E989 *, E1008, R1123	F1, E2, K3, T5, P8
FYQYNPDS	P29000	Acid beta-fructofuranosidase	8	−36.80 ± 0.52	Q886, A989, A959, S960, E989 *, E1016 *, Y1124, K1112, S1127 *	F1, Y2, Q3, Y4, N5, D7, S8
VKVPEPT	P31542	ATP-dependent Clp Ptease ATP-binding subunit ClpA homolog CD4B	7	−31.71 ± 1.87	T667, N671, R729, E989, E1016 *	V1, K2, E5

* Residues of the active site. The main catalytic residues are R791, Y829, H988, E989, H992, E1016, W1090, R1094, H1118, R1127, and R1166-L1667 [UniProt: P12821].

**Table 6:** Top *S. lycopersicum* anti-inflammatory peptides candidate with prediction score results and interacting residues for the target protein.

Peptide Sequence	Accession Number	Protein Name	Residues Number	MMGPBSA (kcal/mol ± SD)	Target Binding Residues	Peptide Binding Residues
PTKGSSVAIFGLGAVG-LAAAEGAR	P28032	Alcohol dehydrogenase 2	24	−50.68 ± 2.3	F476 *, M480	S6, I9
DSSMAGYMSSKKTMEI-NPENSIM	P36181	Heat shock cognate Ptein 80	23	−47.66 ± 1.77	S118, R381, K411, D412, P466, S469	Y7, T13, M14, E15, N20, S21
NQKNLHKRYAYQIVL-QTREMLR	Q84K13	Sine/threonine-protein phosphatase 5	22	−42.12 ± 3.52	D460, F476 *, M480, N482	R8, Y9, Q12, Q16
DKRIFFTNKSYLPSQTP-SGVIR	AAB65766	Lipoxygenase	22	−41.85 ± 1.58	D98, L100, C115, F476, M480, N482	K2, R3, F5, T7
DTQPPRLPTKAVRVTA-EEVR	P54767	Estimate decarboxylase	20	−32.76 ± 1.31	R86, D412, E479	K10, R20
DETPELMPLSHVLATKLGAR	P43280	S-adenosylmethionine synthase 1	20	−32.72 ± 5.92	D412, F476 *,	L9, S10, R20
SAILATPSGERTMTSEQMVY	P08196	Phytoene synthase 1, chloroplastic	20	−32.46 ± 3.72	C115, P466, G470, E479	T6, R11, Y20
CGYSMNSIEGAAVSTIHITPE	ABQ42184	S-adenosylmethionine decarboxylase Penzyme	21	−30.5 ± 0.99	S118, R381 *, Q387	N6, S7, E9

* Residues of the active site. The main catalytic residues are R381, I462, W463, and F476 [UniProt: P35228].

**Table 7:** Top *S. lycopersicum* antimicrobial peptides candidate with prediction score results and interacting residues for each target protein.

Peptide Sequence	Accession Number	Protein Name	Residues Number	MMGPBSA (kcal/mol ± SD)	Target Binding Residues	Peptide Binding Residues
DGPNASYITPAAL	P08196	Phytoene synthase 1, chloroplastic	13	−39.64 ± 0.64	T111, G113, K114, T115 *, N151 *, H181, R187 *, D207, R334, H352, R382	D1, N4, A5, S6, Y7, I8, A11
DFGWGNPIFGGILKAISFTSFGVSVKN	Q6QLX4	Alcohol acyl transferase	27	−34.96 ± 5.57	Y45, E213, H217	P7, K14
DAGASKTYPQQAGTIRKGGHIVIKNRP	Q9AXQ6	Eukaryotic translation initiation factor 5A-1	27	−32.89 ± 3.17	H205, D207, E381, R382, D410	D1, S5, R16, K17,
DKVCVLSCGISTGLGASLNVAKP	P28032	Alcohol dehydrogenase 2	23	−32.30 ± 2.22	V43, V184	S11, L14
DFLIGNTSTGYCAGG-CAAIV	Q40140	Aspartic protease	20	−30.94 ± 2.68	R31, H181, R187 *, E381	D1, T9, A13
Target: *Staphylococcus aureus*; UniProt: Q2FZP6; PDB: 4C12. * Residues of the active domain G110–S116, N151, T152–T513, S179, and R187.						
DAGASKTYPQQAGTIRKGGHIVIKNRP	Q9AXQ6	Eukaryotic translation initiation factor 5A-1	27	−32.07 ± 4.96	D5(A), E7(A), K8(A), R10(A), M1(B)	D1, A2, R16, G18, G19, H20
Target: *Helicobacter pylori*; UniProt: O25608; PDB: 3QDL. * Residues of the active domain G150–G155. (A) and (B) denote the different chain.						
DHVGFSCSTSGGAASRGILGPFGVIVIA	P29000	Acid beta-fructofuranosidase	28	−49.54 ± 4.09	T206(A), D63(B), E146(B), N148(B)	S8, T9, G12, R16, I18, G20
DFLIGNTSTGYCAG-GCAAIV	Q40140	Aspartic protease	20	−47.72 ± 1.56	T206(A), A208(A), Y67(B), E146(B), G209(B), N210(B), V211(B), D213(B)	D1, S8, T9, Y11, A13, G14
DAGASKTYPQQAGTIRKNGYIVIKGRP	Q9AXQ3	Eukaryotic translation initiation factor 5A-4	27	−45.81 ± 5.54	D63(A) H116 * (A), D118 * (A), R205(A), N210(A), T206(B), A208(B)	D1, A2 G3, A4, K6, Y8, Q10, K17
DKVCVLSCGISTGLGASLNVAKP	P28032	Alcohol dehydrogenase 2	23	−39.7 ± 2.04	T206(A), D63(B), E146(B), N148(B)	C8, T12, S17, N19, K22
DAGASKTYPQQAGTIRKGGHIVIKNRP	Q9AXQ6	Eukaryotic translation initiation factor 5A-1	27	−30.6 ± 1.28	L203(A), D247(A), N254(A), D63(B), D117(B), D118 * (B), E146(B)	D1, K6, T7, K17, N25, R26
Target: *Pseudomonas aeruginosa*; UniProt: D1MEN9; PDB: 6EW3. * Residues of the active domain H114, H116, D118, H153, H179, C198, and H240. (A) and (B) denote the different chains.						
DHVGFSCSTSGGAA-SRGILGPFGVIVIA	P29000	Acid beta-fructofuranosidase	28	−45.95 ± 3.77	E87 *, D130 *, D178 *, H185, T189, R192, K205	D1, H2, R16, I27
DGPNASYITPAAL	P08196	Phytoene synthase 1, chloroplastic	13	−44.92 ± 1.42	D47 *, D49 *, E87 *, W112, R192, K205	D1, S6
DFLIGNTSTGYCAG-GCAAIV	Q40140	Aspartic protease	20	−33.77 ± 3.73	D47 *, E87, R105, Q126, W129, D130 *, D171, D182	D1, L3, E5, N6, T7, Y11
DKVCVLSCGISTGLGASLNVAKP	P28032	Alcohol dehydrogenase 2	23	−32.74 ± 5.71	D128, D178, D182, H185, R192	S11, L14, G15, L18, N19
Target: *Salmonella enterica* subsp. enterica serovar Typhimurium str. LT2; UniProt: Q8ZPX9, PDB: 6FZB. * Residues of the active domain S36, S46, D47, D49, E87, D130, W173–D178, H185, K205, and Y231.						
DAGASKTYPQQAGTIRKGGHIVIKNRP	Q9AXQ6	Eukaryotic translation initiation factor 5A-1	27	−32.07 ± 1.49	D311, E676 *, G678 *, T679 *, I742, N743	S5, T7, Y8, K24, R26
DKVCVLSCGISTGLGASLNVAKP	P28032	Alcohol dehydrogenase 2	23	−31.44 ± 2.67	Y180	T12, N19
Target: *Acinetobacter baumannii*; UniProt: G1C794, PDB: 3UDF. * Residues of the active domain G321–A737.						

## Data Availability

Data will be made available on request.

## Supplementary Materials

The following supporting information can be downloaded at https://www.mdpi.com/article/10.3390/biom14080930/s1. Figure S1. Images of docking poses of ACP peptides in complex with Aminopeptidase N (PDB ID: 4FYQ) overlayed over co-crystallized ligand (PDB ID: 4FYS), and LigPlot+ analysis. Figure S2. Images of docking poses of ADP peptides in complex with Dipeptidyl Peptidase IV (PDB ID: 2ONC) overlayed over co-crystallized ligand (PDB ID: 2ONC), and LigPlot+ analysis. Figure S3. Images of docking poses of AHP peptides in complex with Angiotensin Converting Enzyme (PDB ID: 1O86) overlayed over co-crystallized ligand (PDB ID: 1O86), and LigPlot+ analysis. Figure S4. Images of docking poses of AIP peptides in complex with Nitric oxide synthase (PDB ID: 3E7G) overlayed over co-crystallized ligand (PDB ID: 3E7G), and LigPlot+ analysis. Figure S5. Images of docking poses of AMP peptides in complex with Penicillin-binding protein 1a (PDB ID: 3UDF) of *Acinetobacter baumanii* overlayed over co-crystallized ligand (PDB ID: 3UDF), and LigPlot+ analysis. Figure S6. Images of docking poses of AMP peptides in complex with Oxygen-intensive NADPH nitroreductase (PDB ID: 3QDL) of *Helicobacter pylori* overlayed over co-crystallized ligand (PDB ID: 3QDL), and LigPlot+ analysis. Figure S7. Images of docking poses of AMP peptides in complex with UDP-N-acetylmuramoyl-L-alanyl-D-glutamate-L-lysine ligase (PDB ID: 4C12) of *Staphylococcus aureus* overlayed over co-crystallized ligand (PDB ID: 4C12), and LigPlot+ analysis. Figure S8. Images of docking poses of AMP peptides in complex with Streptomycin 3″-adenylyltransferase (PDB ID: 6FZB) of *Salmonella enterica* overlayed over co-crystallized ligand (PDB ID: 6FZB), and LigPlot+ analysis. Figure S9. Images of docking poses of AMP peptides in complex with Metallo-beta-lactamase type 2 (PDB ID: 6EW3) of *Pseudomonas aeruginosa* overlayed over co-crystallized ligand (PDB ID: 3UDI), and LigPlot+ analysis. Supplementary Data File: Supplemental data of this manuscript.
