# Supplementary material for: A Novel Workflow for In Silico Prediction of Bioactive Peptides: An Exploration of Solanum lycopersicum By-Products
Source: Biomolecules. 2024 Jul 31;14(8):930. doi: 10.3390/biom14080930 (PMC11352670; doi:10.3390/biom14080930)
Supplement: Supplementary file 1 [file biomolecules-14-00930-s001.zip › Supplementary_data-files/README.pdf]

Supplemetary data-files to “A Novel Workflow for In Silico Prediction of Bioactive Pep-tides: an Exploration on Solanum Lycopersicum By-Products”.

Francesco Morena [1,*], Chiara Cencini [1], Eleonora Calzoni [1], Sabata Martino [1,2,‡], and Carla Emiliani [1,2,‡,*]

[1] Department of Chemistry, Biology and Biotechnology, Biochemistry and Molecular Biology Section, University of Perugia, Via del Giochetto, 06123 Perugia, Italy

[2] Centro di Eccellenza su Materiali Innovativi Nanostrutturati (CEMIN), University of Perugia, Via del Giochetto, 06123 Perugia, Italy

Each folder of bioactivity (ACP, ADP, AHP, AIP, and AMP) is composed by thew following file which correspond to the different steps in the workflow:

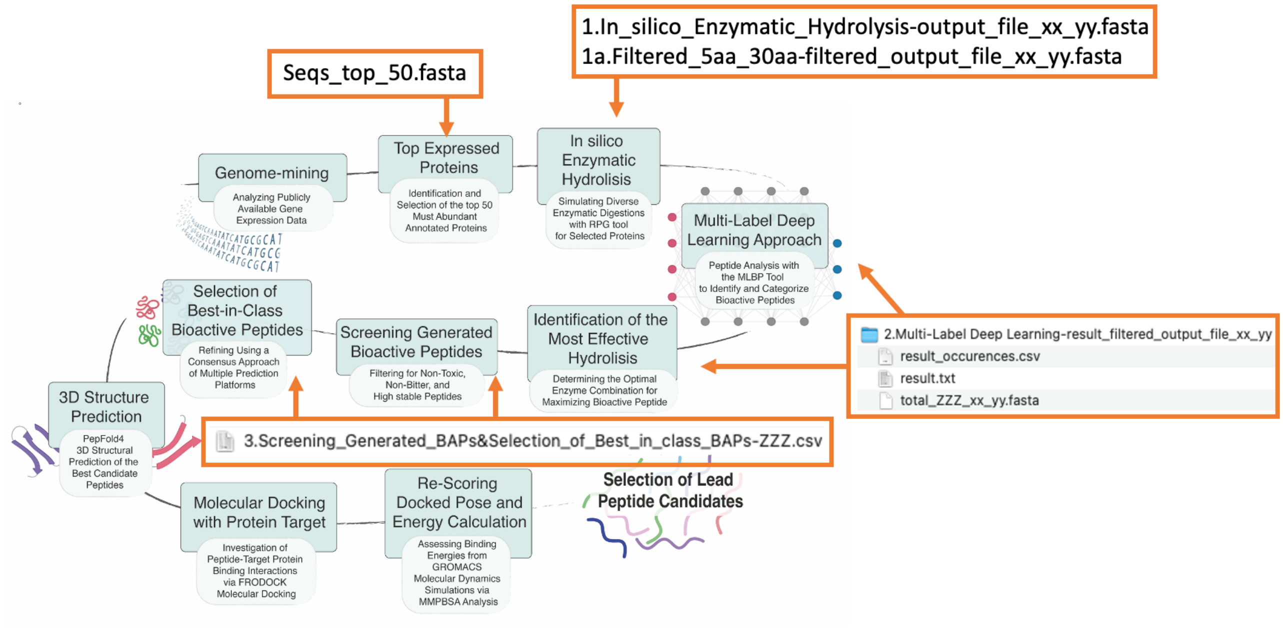

The variables in the figure include:

xx = first enzyme number used (refer to RPG available enzyme, https://rapid-peptide-generator.readthedocs.io/en/latest/enzymes.html)

yy = second enzyme number used (refer to RPG available enzyme, https://rapid-peptide-generator.readthedocs.io/en/latest/enzymes.html)

ZZZ = bioactive class identified by MLBP (ACP, ADP, AHP, AIP, and AMP)
