# Supplementary material for: A Novel Workflow for In Silico Prediction of Bioactive Peptides: An Exploration of Solanum lycopersicum By-Products
Source: Biomolecules. 2024 Jul 31;14(8):930. doi: 10.3390/biom14080930 (PMC11352670; doi:10.3390/biom14080930)

Supplementary file

A Novel Workflow for In Silico Prediction of Bioactive Pep-tides: an Exploration of Solanum Lycopersicum By-Products

Francesco Morena [1,*], Chiara Cencini [1], Eleonora Calzoni [1], Sabata Martino [1,2,‡], and Carla Emiliani [1,2,‡,*]

[1] Department of Chemistry, Biology and Biotechnology, Biochemistry and Molecular Biology Section, University of Perugia, Via del Giochetto, 06123 Perugia, Italy

[2] Centro di Eccellenza su Materiali Innovativi Nanostrutturati (CEMIN), University of Perugia, Via del Giochetto, 06123 Perugia, Italy

Contact info

*Correspondence: francesco.morena@unipg.it, carla.emiliani@unipg.it

‡ These authors contributed equally to this work

Content

Figure S1: Images of docking poses of ACP peptides in complex with Aminopeptidase N (PDB ID: 4FYQ) overlayed over co-crystallized ligand (PDB ID: 4FYS), and LigPlot+ analysis.

Figure S2: Images of docking poses of ADP peptides in complex with Dipeptidyl Peptidase IV (PDB ID: 2ONC) overlayed over co-crystallized ligand (PDB ID: 2ONC), and LigPlot+ analysis.

Figure S3: Images of docking poses of AHP peptides in complex with Angiotensin Converting Enzyme (PDB ID: 1O86) overlayed over co-crystallized ligand (PDB ID: 1O86), and LigPlot+ analysis.

Figure S4: Images of docking poses of AIP peptides in complex with Nitric oxide synthase (PDB ID: 3E7G) overlayed over co-crystallized ligand (PDB ID: 3E7G), and LigPlot+ analysis.

Figure S5: Images of docking poses of AMP peptides in complex with Penicillin-binding protein 1a (PDB ID: 3UDF) of *Acinetobacter baumanii* overlayed over co-crystallized ligand (PDB ID: 3UDF), and LigPlot+ analysis.

Figure S6: Images of docking poses of AMP peptides in complex with Oxygen-intensive NADPH nitroreductase (PDB ID: 3QDL) of *Helicobacter pylori* overlayed over co-crystallized ligand (PDB ID: 3QDL), and LigPlot+ analysis.

Figure S7: Images of docking poses of AMP peptides in complex with UDP-N-acetylmuramoyl-L-alanyl-D-glutamate-L-lysine ligase (PDB ID: 4C12) of *Staphylococcus aureus* overlayed over co-crystallized ligand (PDB ID: 4C12), and LigPlot+ analysis.

Figure S8: Images of docking poses of AMP peptides in complex with Streptomycin 3″-adenylyltransferase (PDB ID: 6FZB) of *Salmonella enterica* overlayed over co-crystallized ligand (PDB ID: 6FZB), and LigPlot+ analysis.

Figure S9: Images of docking poses of AMP peptides in complex with Metallo-beta-lactamase type 2 (PDB ID: 6EW3) of *Pseudomonas aeruginosa* overlayed over co-crystallized ligand (PDB ID: 3UDI), and LigPlot+ analysis.

Figure S1.

Images of the best docking poses of ACP peptides in complex with Aminopeptidase N (PDB ID:4FYQ) overlayed over co-crystallized ligand (PDB ID: 4FYS) and LigPlot+ analysis. The amino acid residues involved in hydrophobic interactions are represented by red arcs, while hydrogen bonds are depicted as green dashed lines with their bond lengths specified.

ACP peptide: MWKLPMFGCT

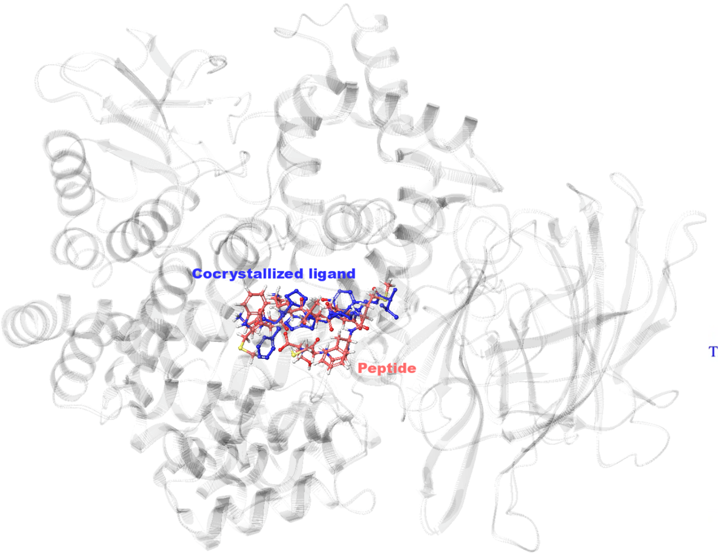

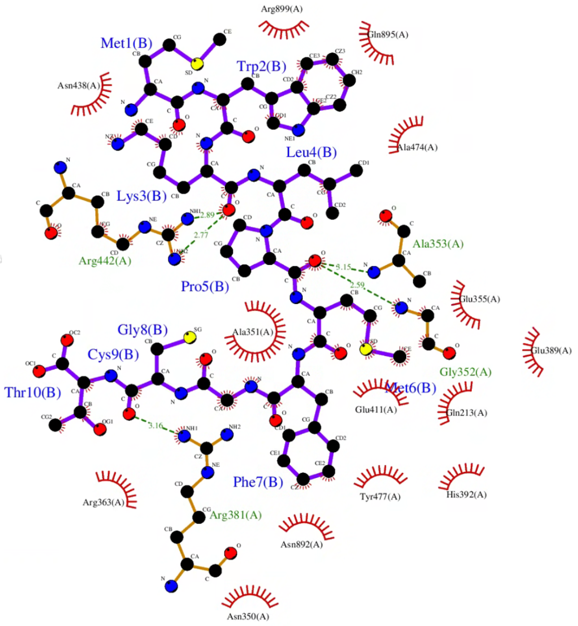

ACP peptide: WKLPM

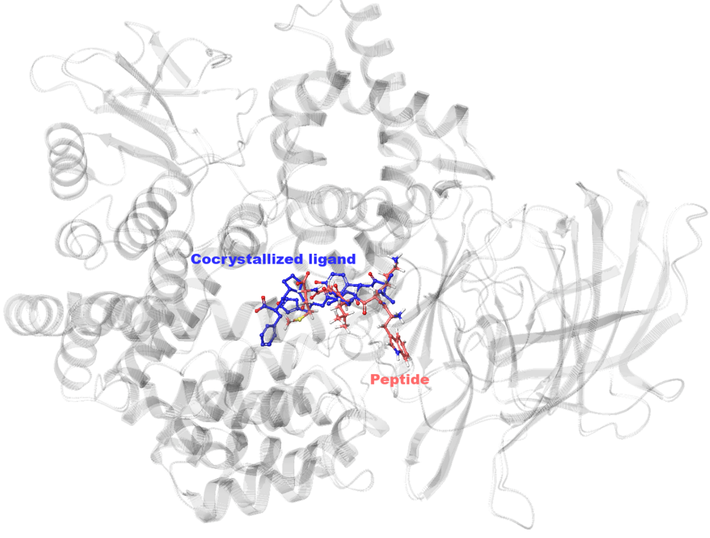

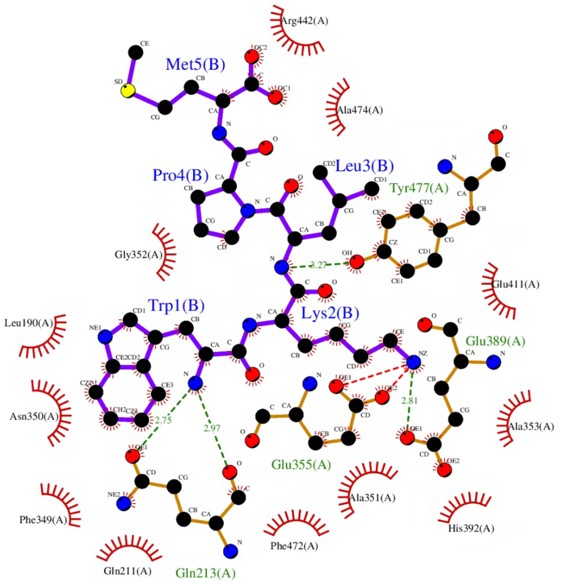

Figure S2.

Images of the best docking poses of ADP peptides in complex with Dipeptidyl Peptidase IV (DPP-IV) (PDB ID: 2ONC) overlayed over co-crystallized ligand (PDB ID: 2ONC) and LigPlot+ analysis. The amino acid residues involved in hydrophobic interactions are represented by red arcs, while hydrogen bonds are depicted as green dashed lines with their bond lengths specified.

ADP peptide: DLLNIFE

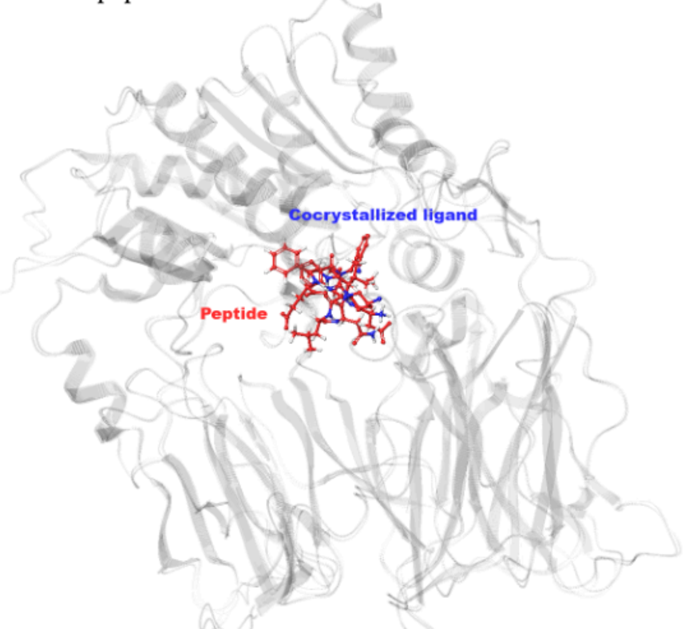

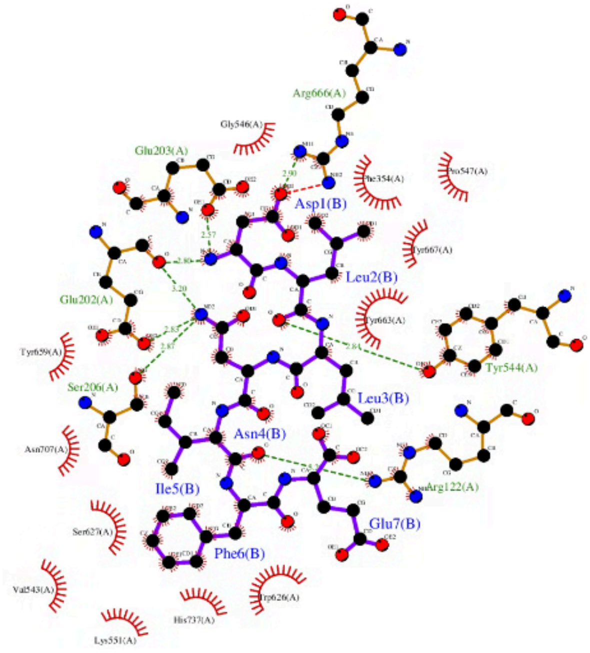

ADP peptide: LMAALNLVG

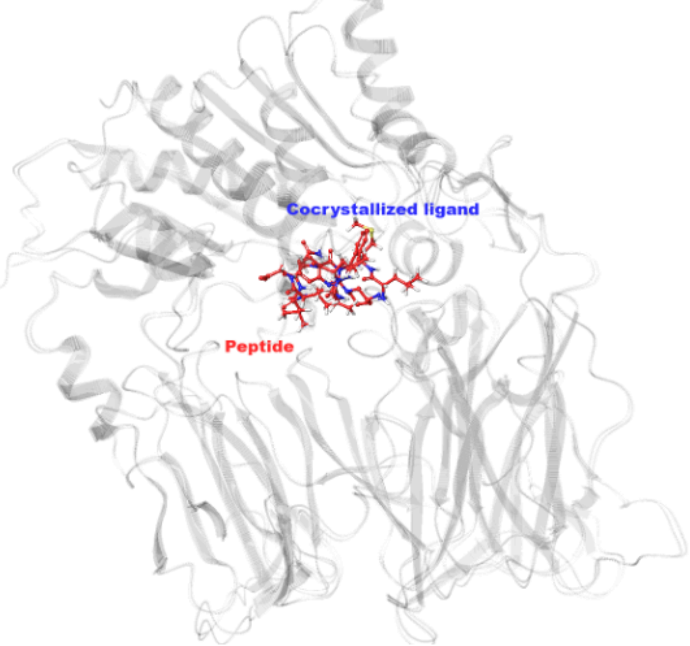

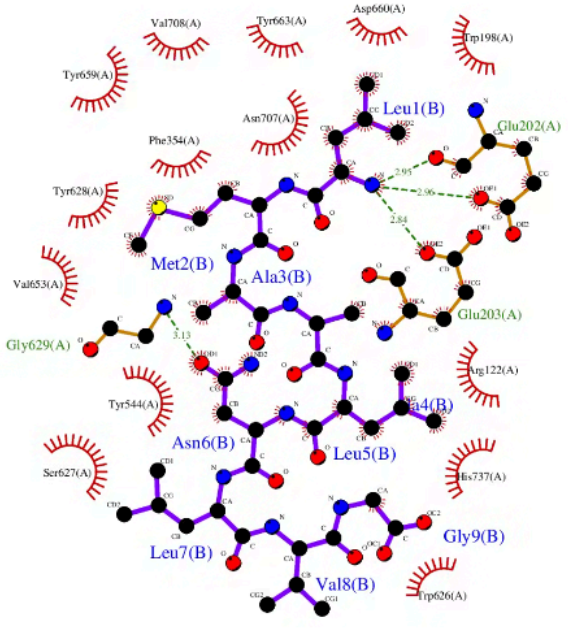

ADP peptide: HWLNTHAVIE

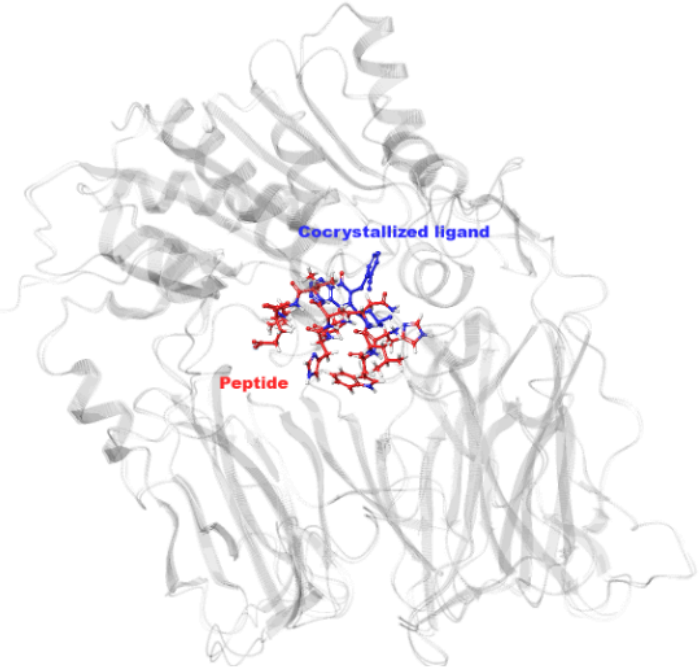

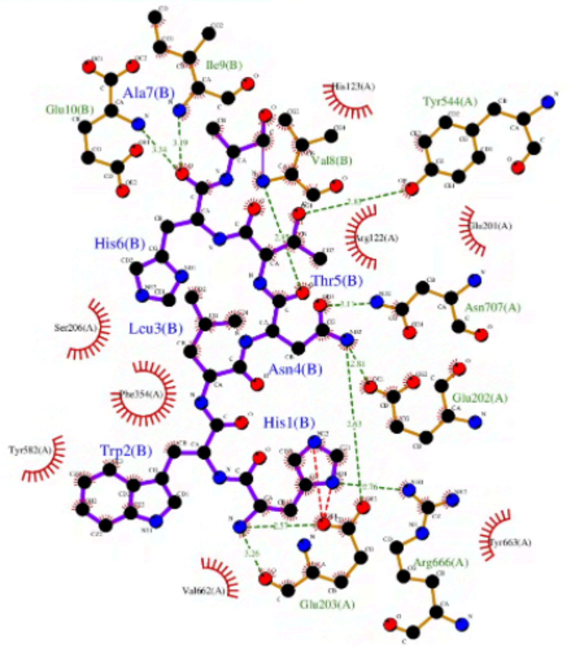

ADP peptide: TFAFQAE

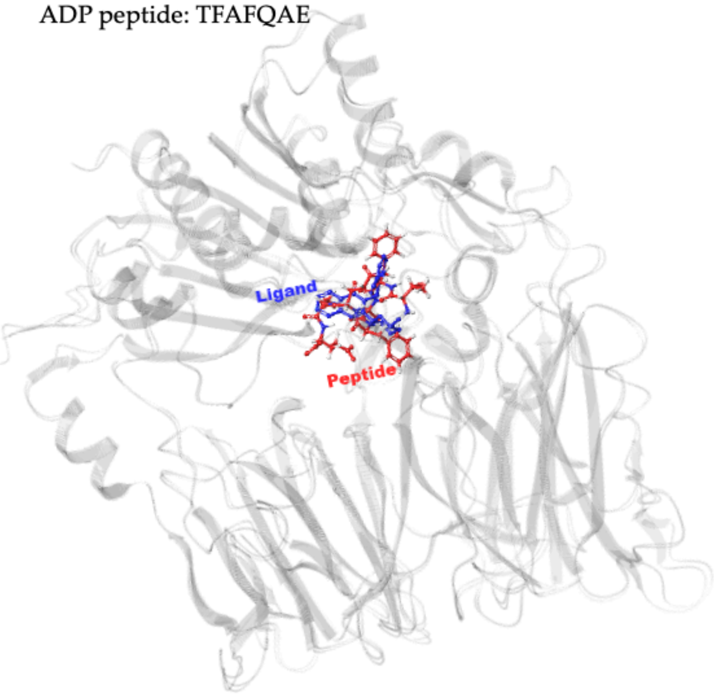

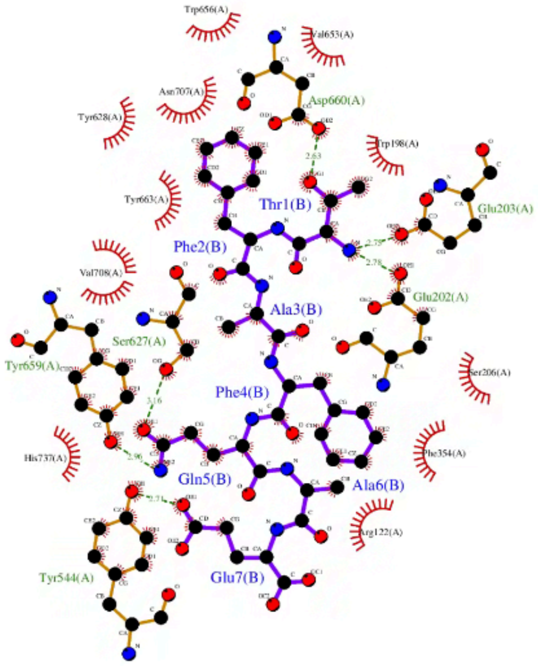

ADP peptide: QFVTFMK

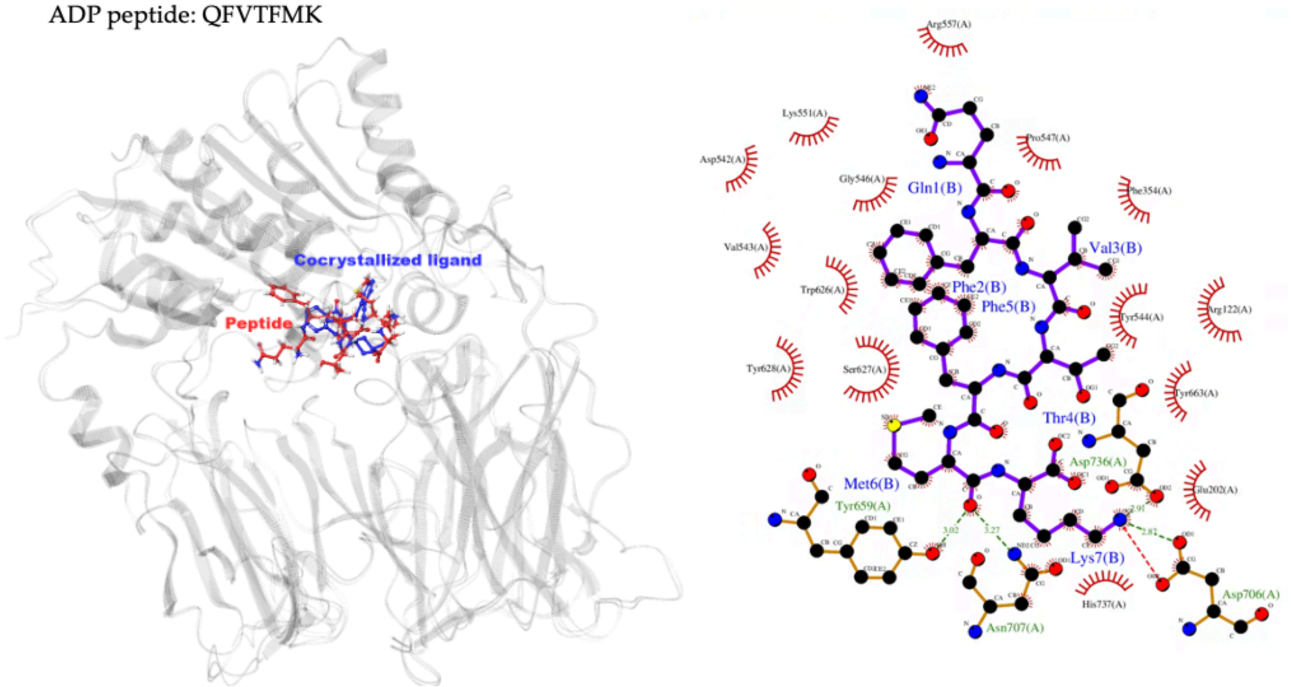

ADP peptide: QEFAHDFQAY

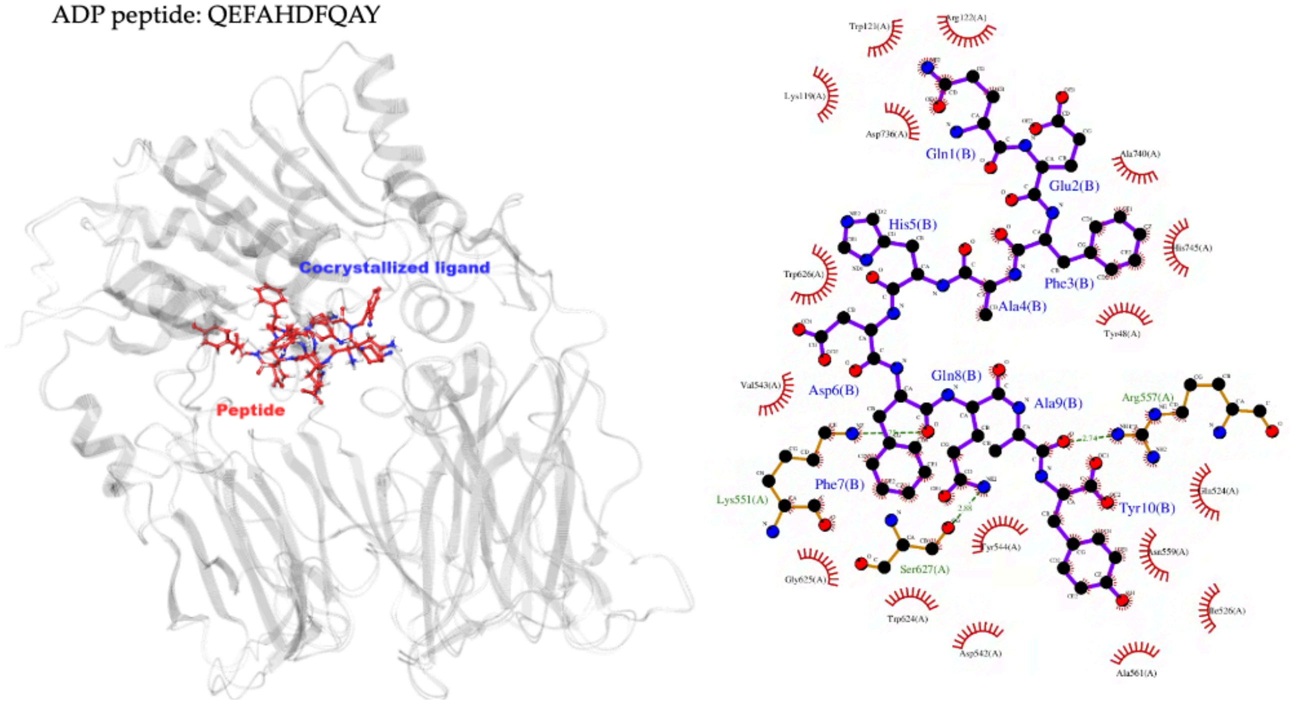

ADP peptide: LPHPDGDQFG

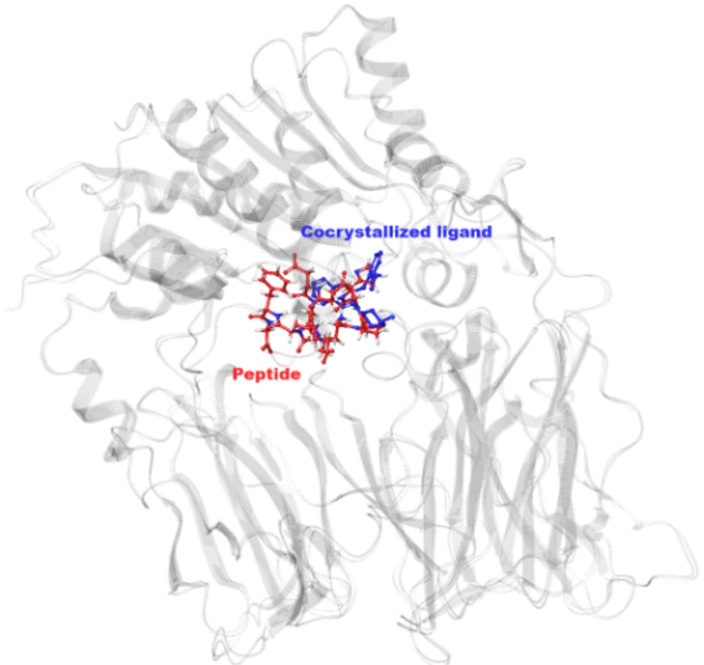

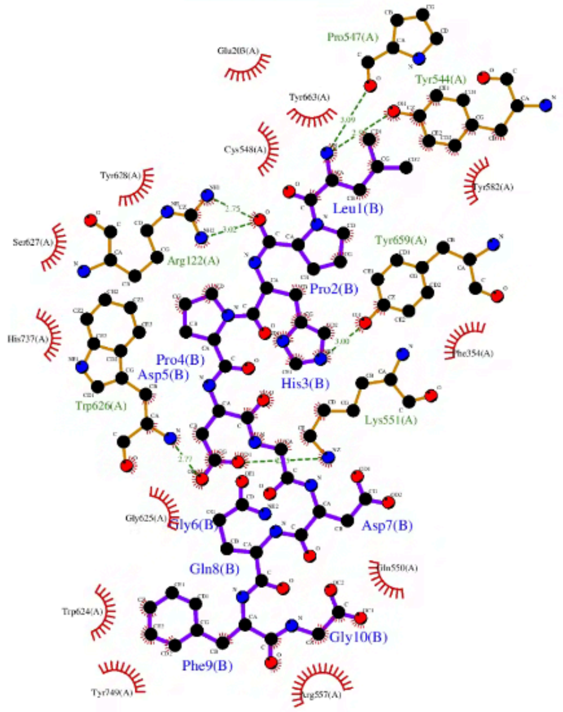

ADP peptide: GWAPQVLLLS

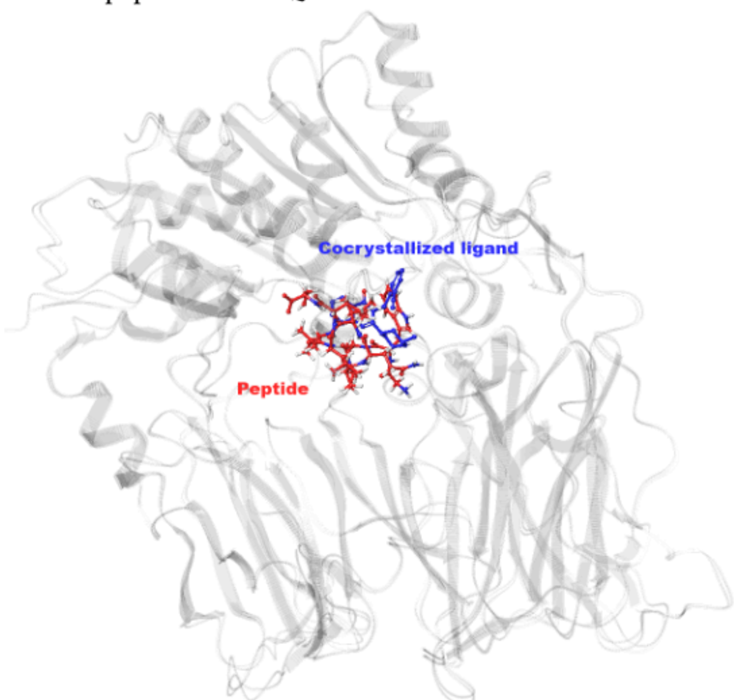

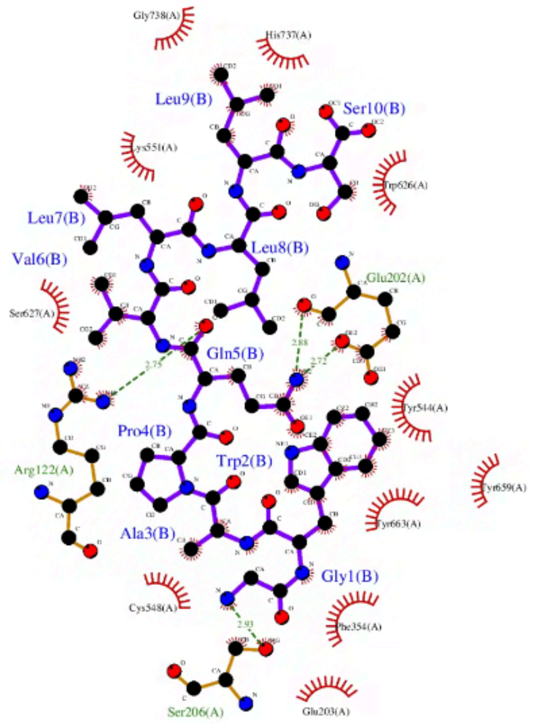

ADP peptide: LAQNNVMFE

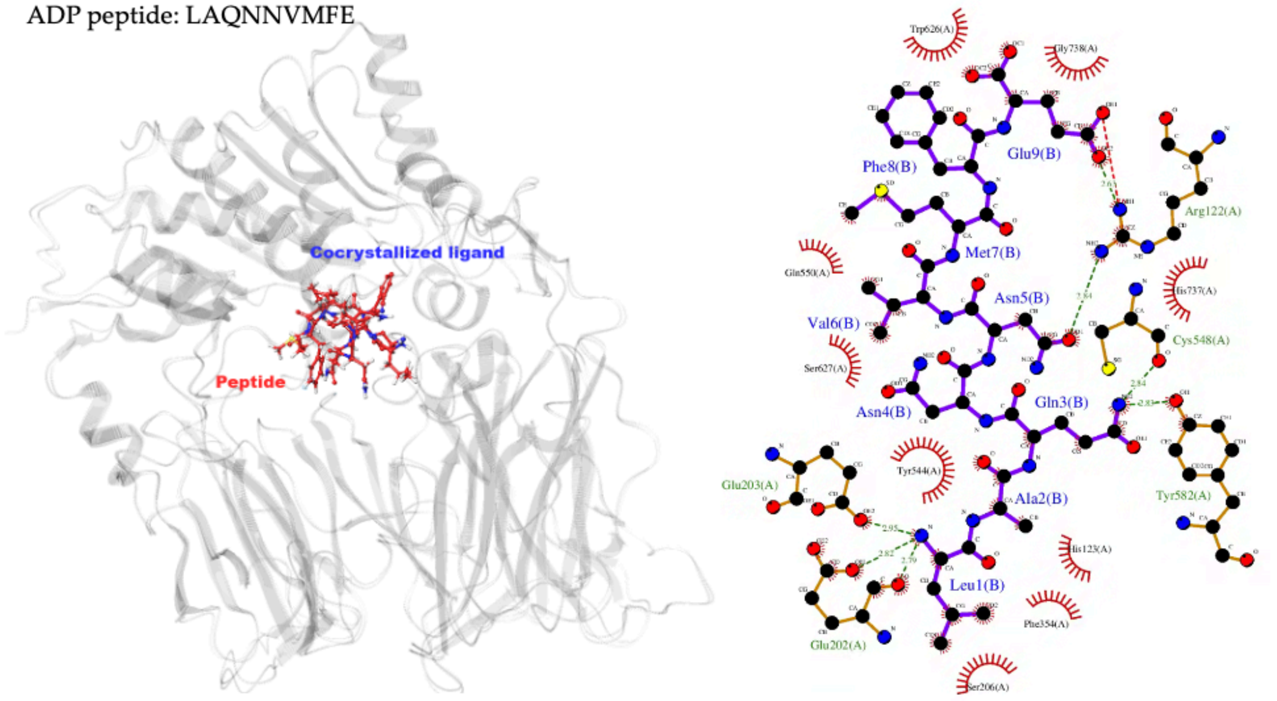

ADP peptide: EMIWDLLVS

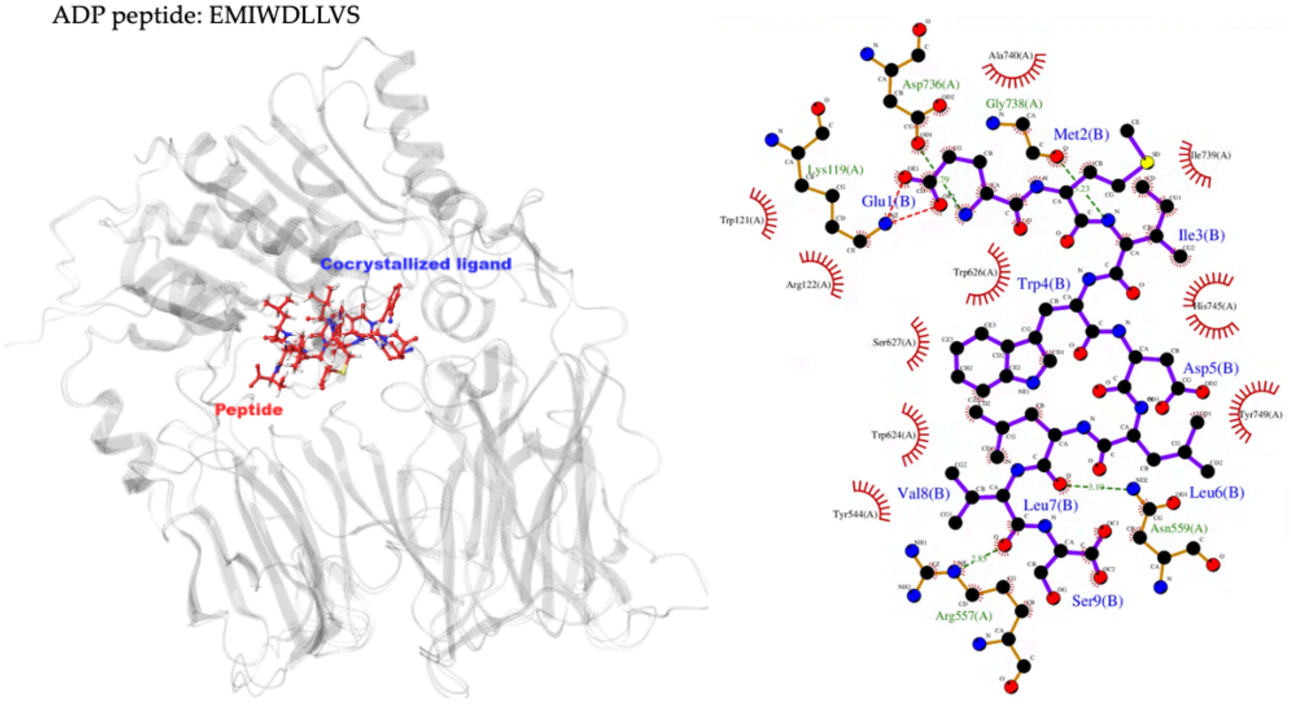

Figure S3

Images of the best docking poses of AHP peptides in complex with Angiotensin Converting Enzyme (ACE) (PDB ID: 1O86) overlayed over co-crystallized ligand (PDB ID: 1O86), and LigPlot+ analysis. The amino acid residues involved in hydrophobic interactions are represented by red arcs, while hydrogen bonds are depicted as green dashed lines with their bond lengths specified.

AHP peptide: MEMGESP

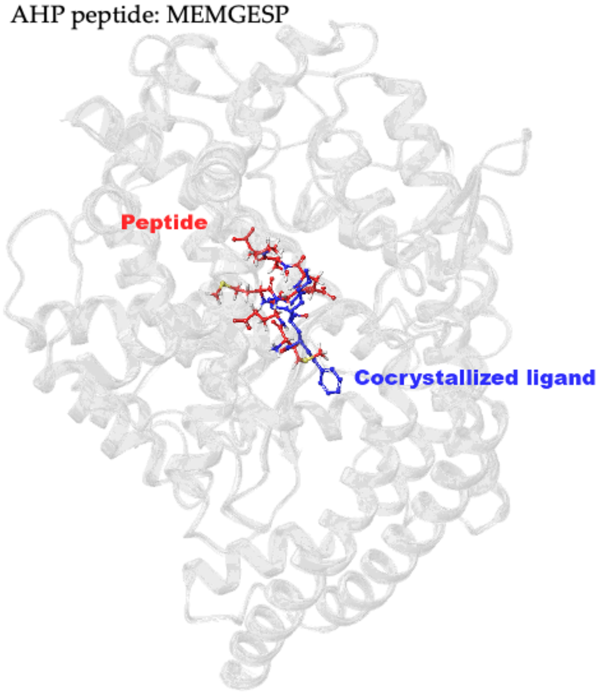

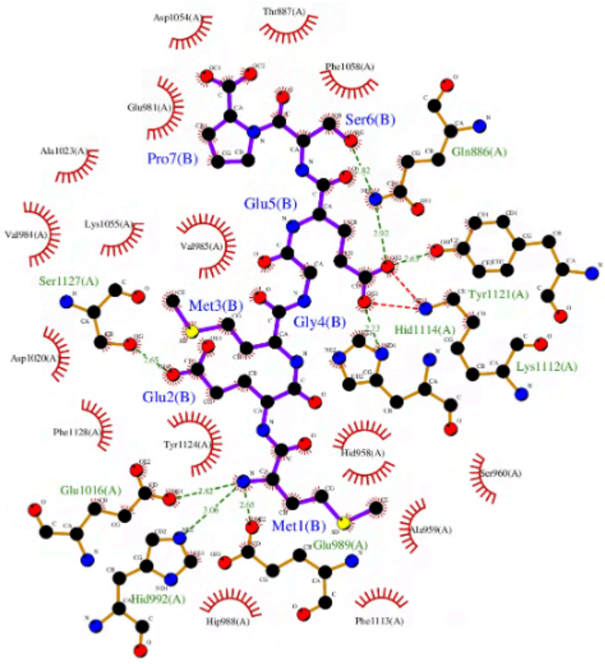

AHP peptide: VEMQDVKYP

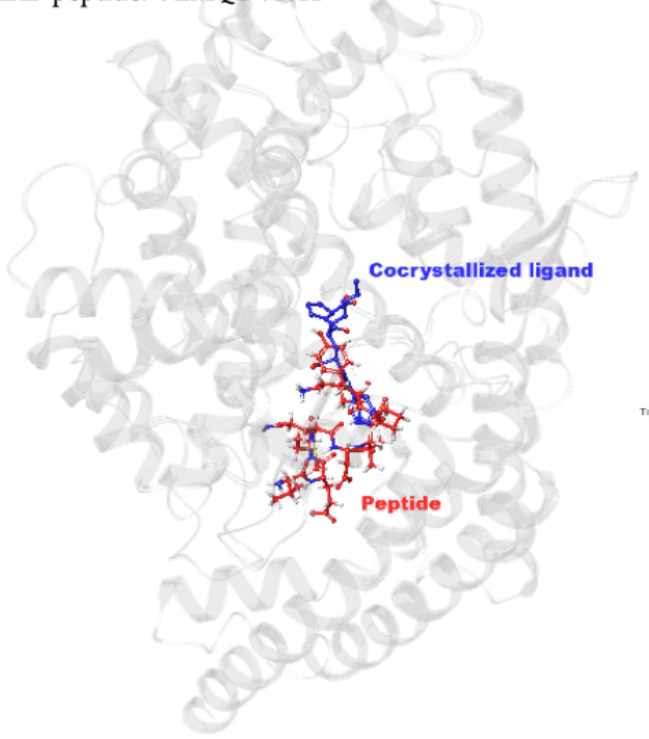

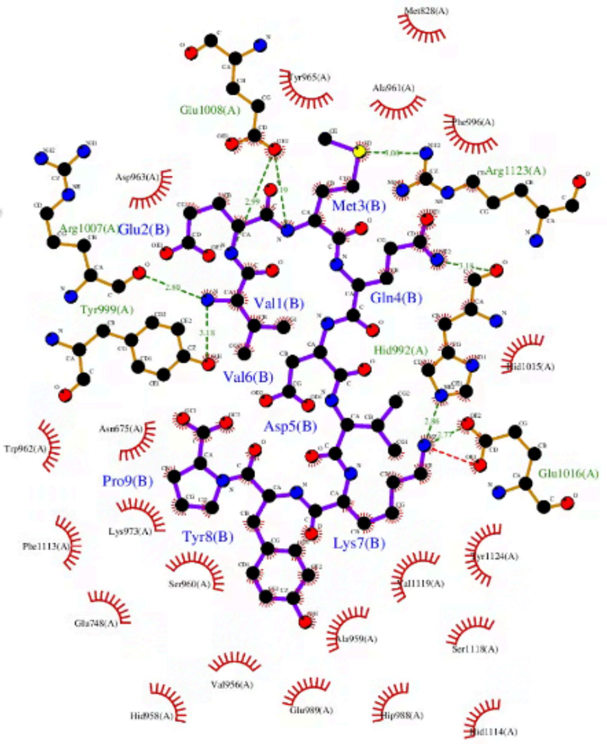

AHP peptide: MEEVDVAPPQK

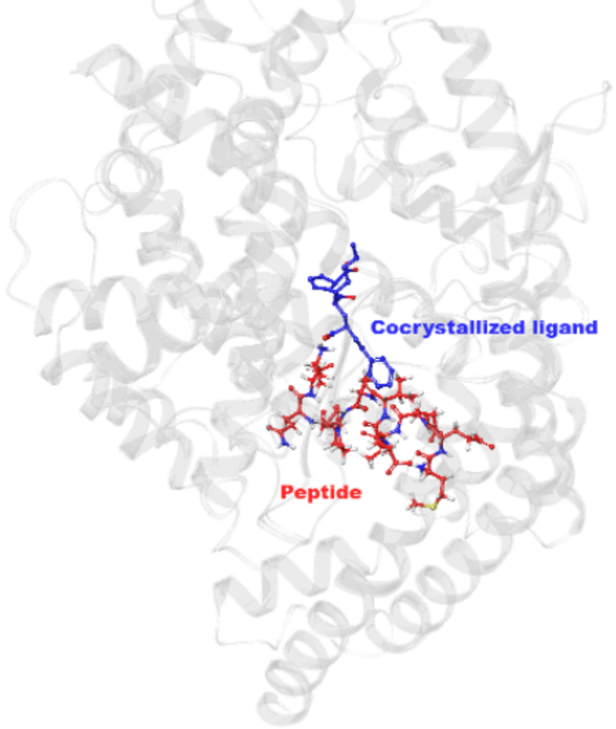

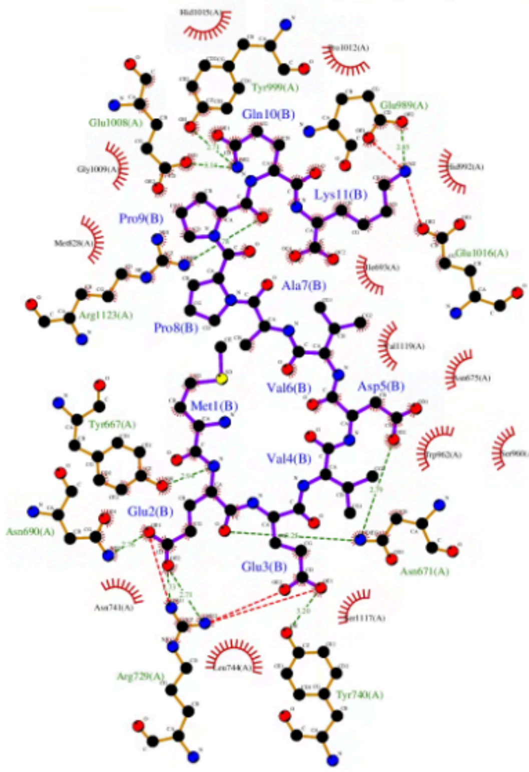

AHP peptide: ANQPLPDDDDEA

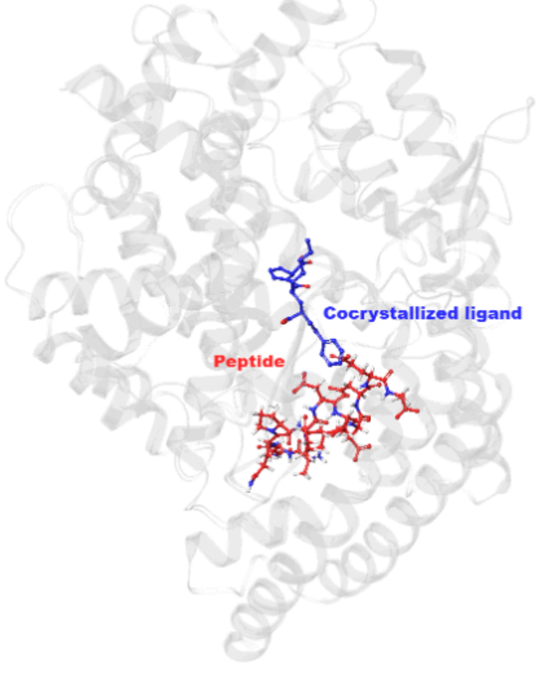

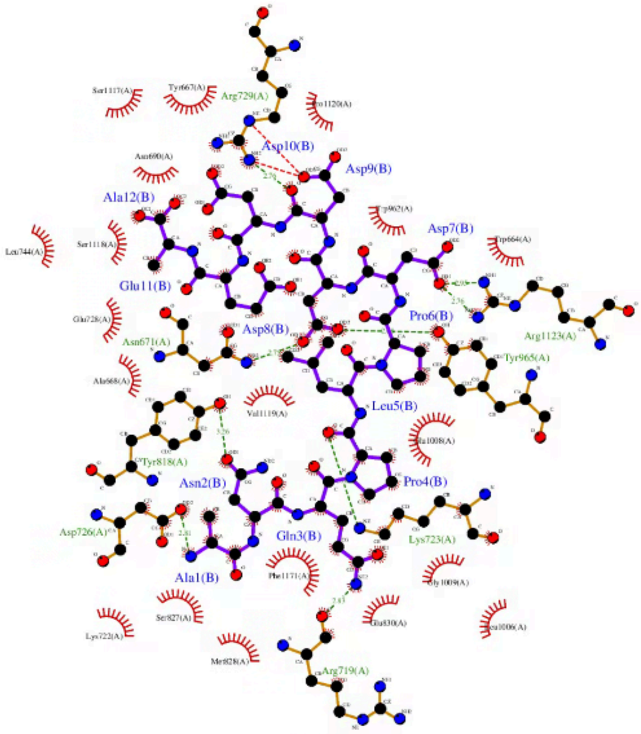

AHP peptide: VIPKENN

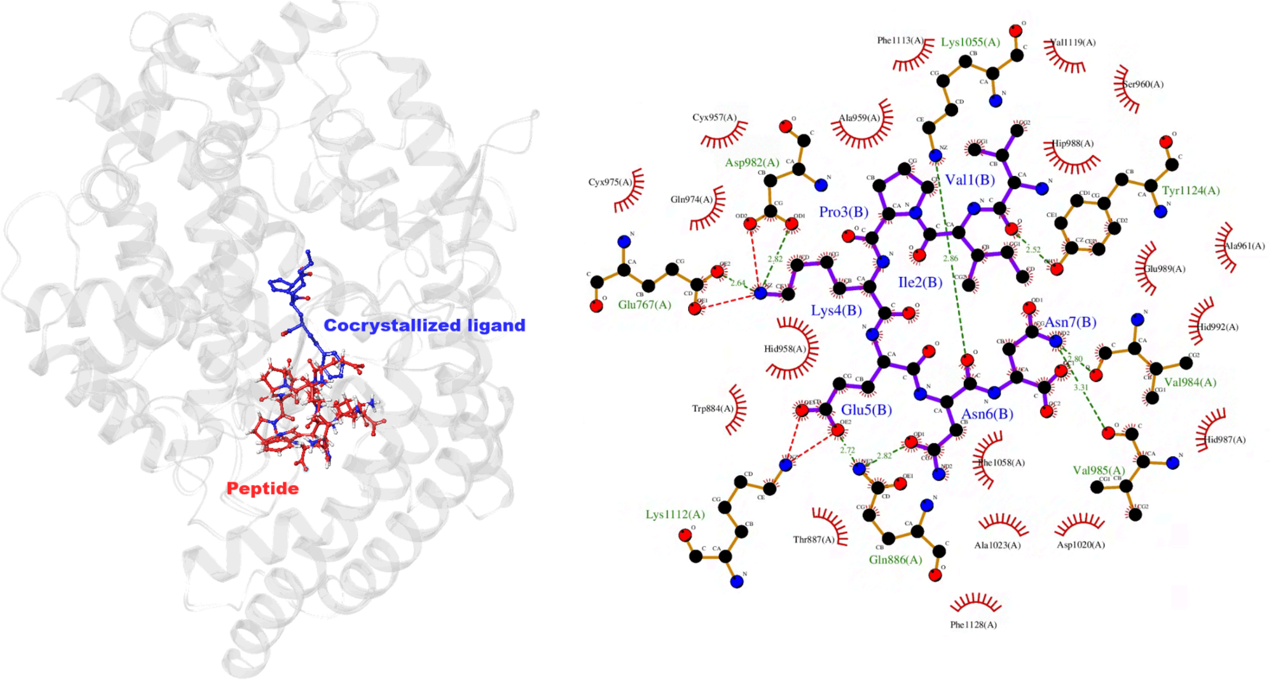

AHP peptide: IDWKETPEPH

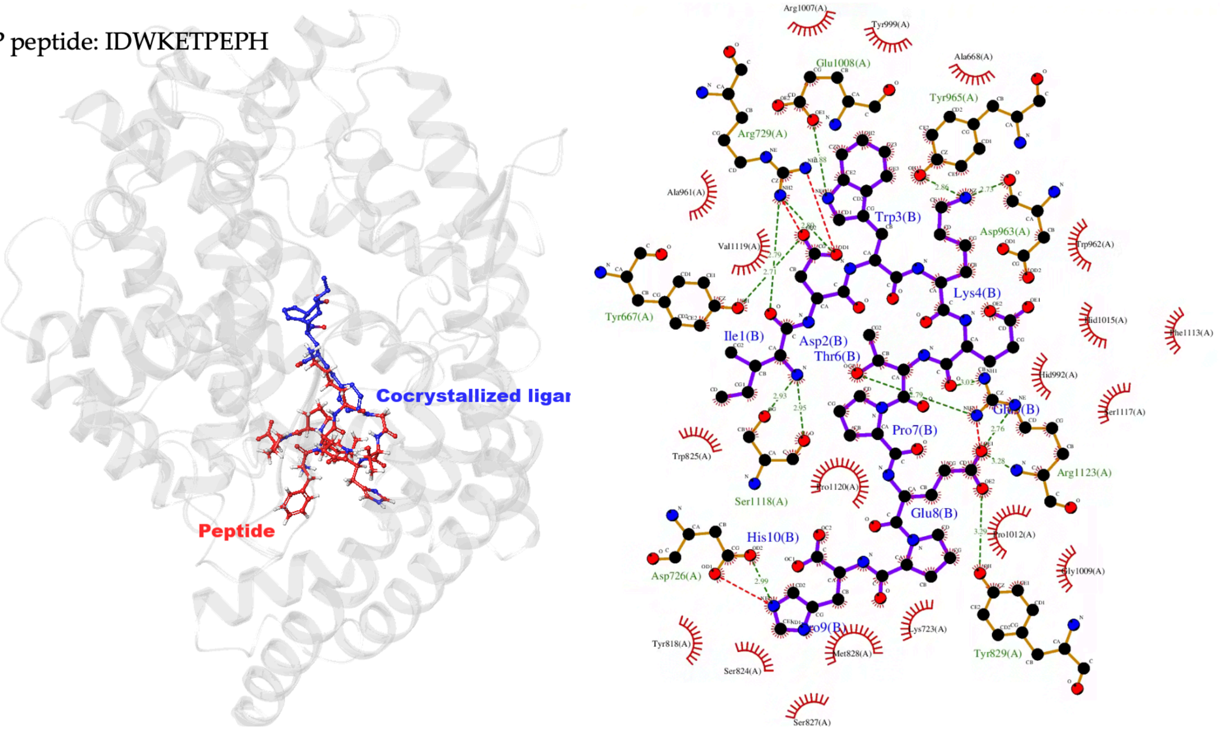

AHP peptide: FEKGTHIPP

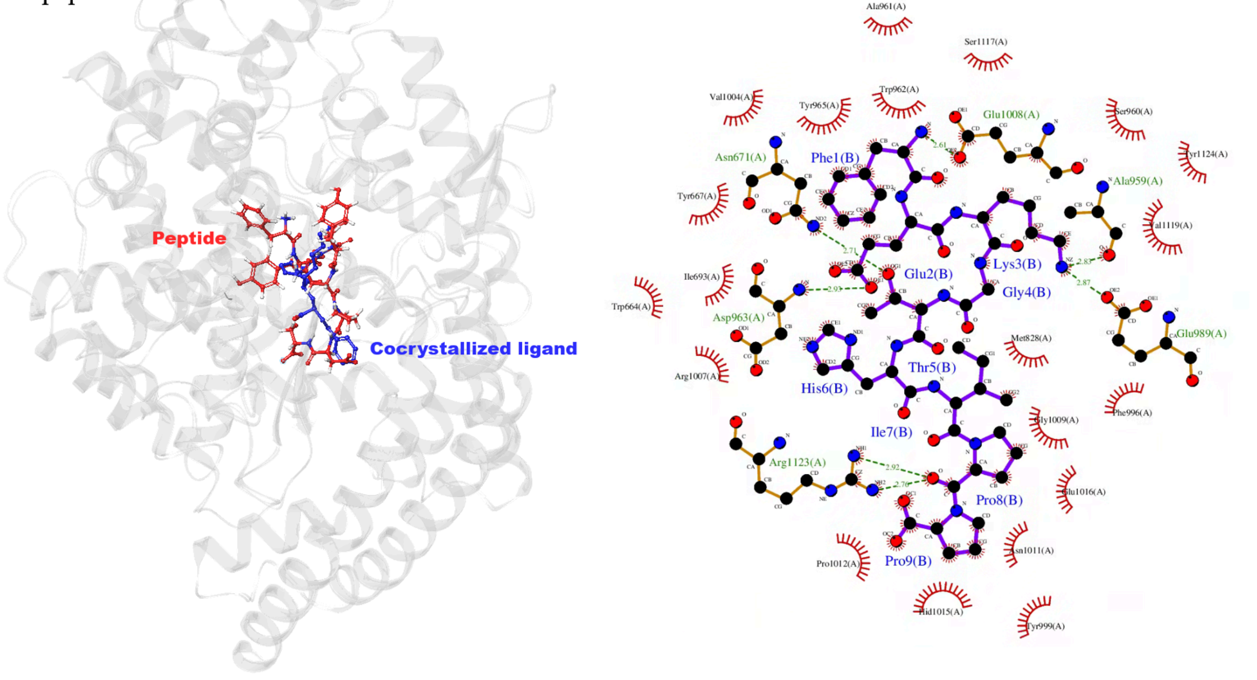

AHP peptide: FYQYNPDS

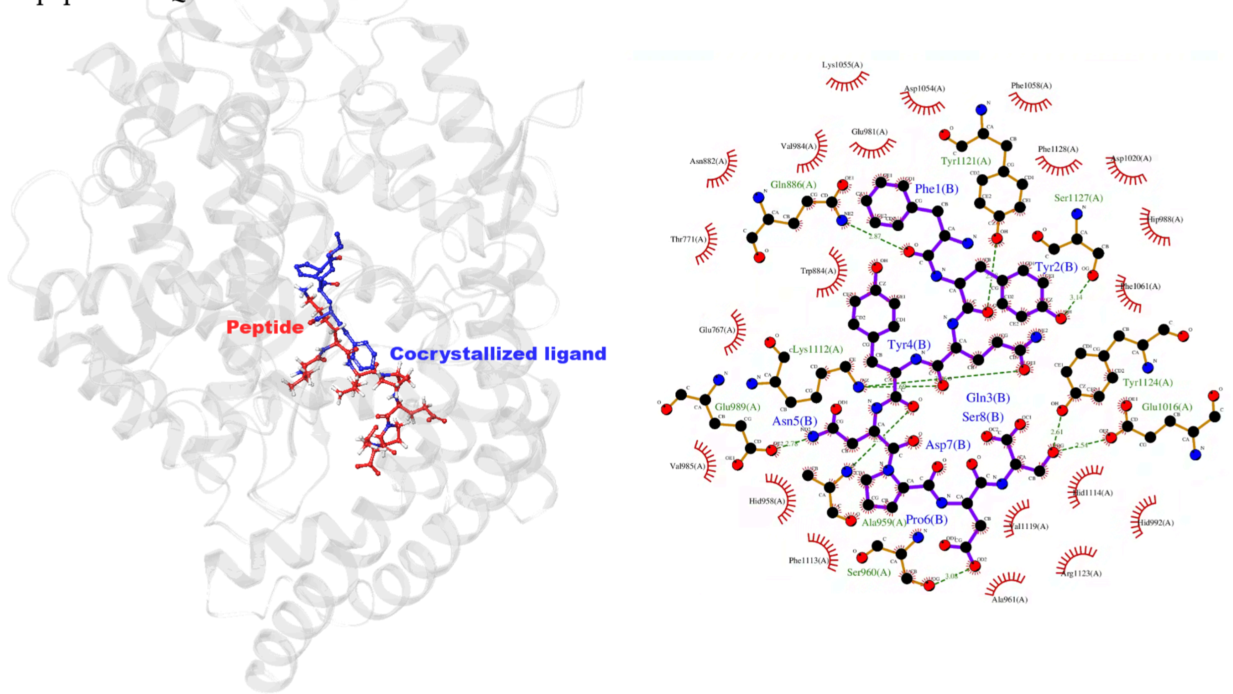

AHP peptide: VKVPEPT

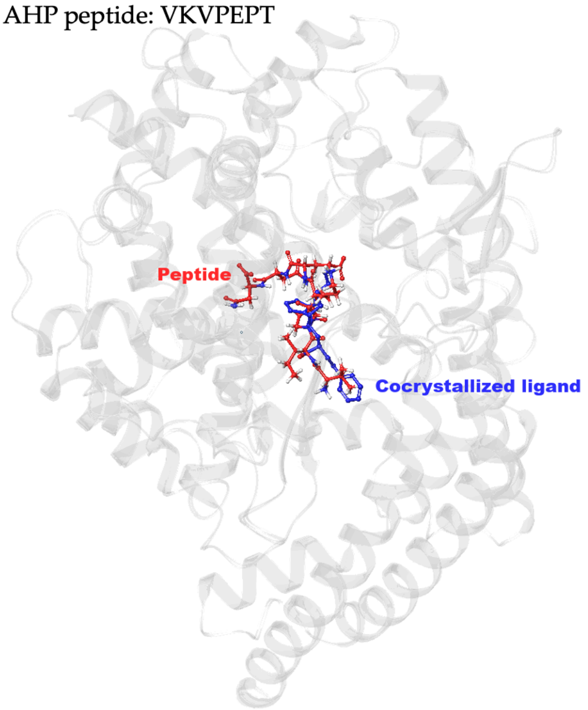

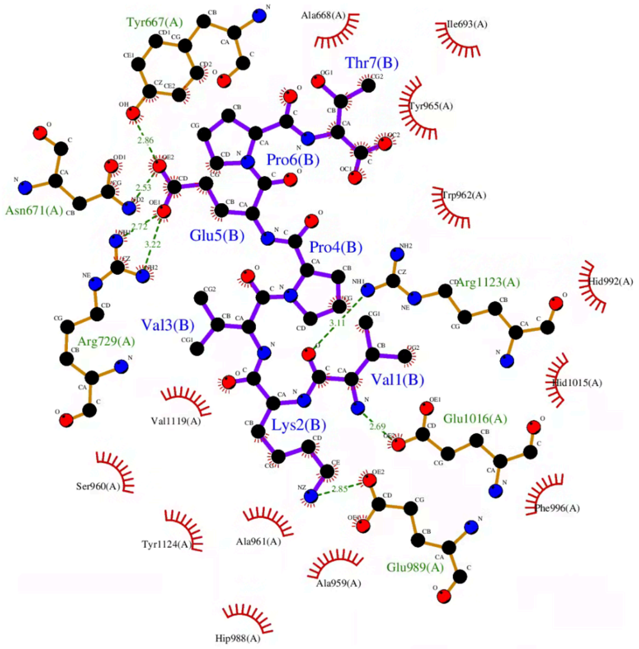

Figure S4

Images of the best docking poses of AIP peptides in complex with inducible Nitric oxide synthase (iNOS) (PDB ID: 3E7G) overlayed over co-crystallized ligand (PDB ID: 3E7G), and LigPlot+ analysis. The amino acid residues involved in hydrophobic interactions are represented by red arcs, while hydrogen bonds are depicted as green dashed lines with their bond lengths specified.

AIP peptide: PTKGSSVAIFGLGAVGLAAAEGAR

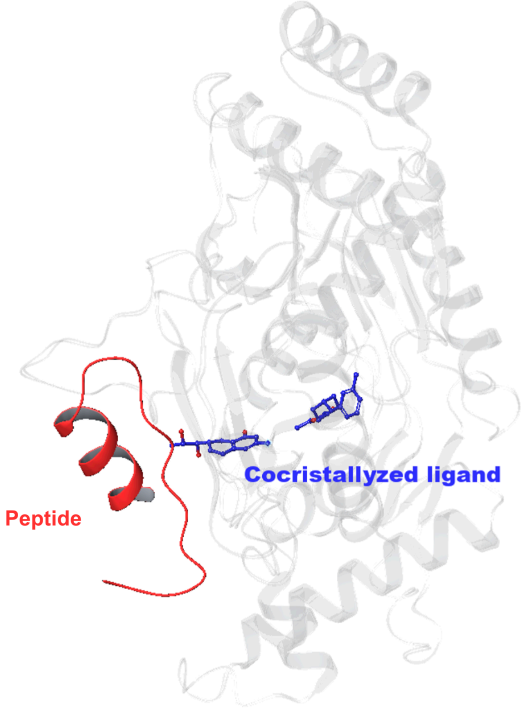

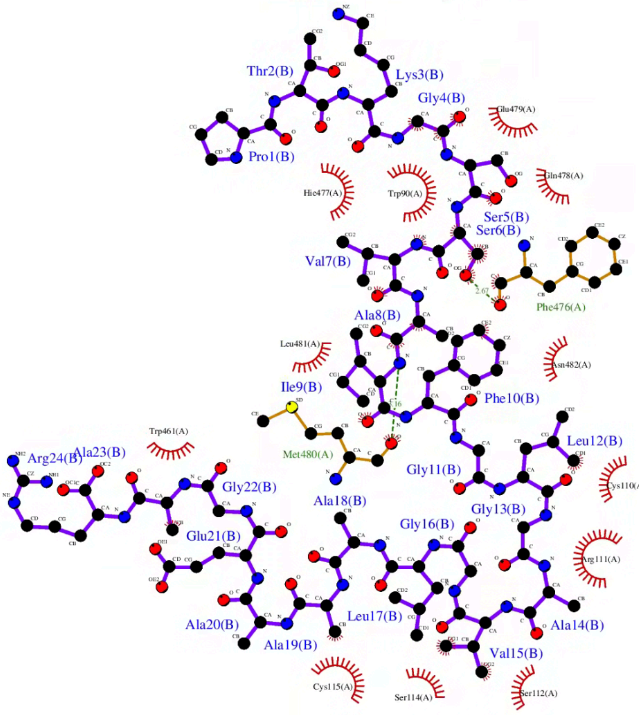

AIP peptide: DSSMAGYMSSKKTMEINPENSIM

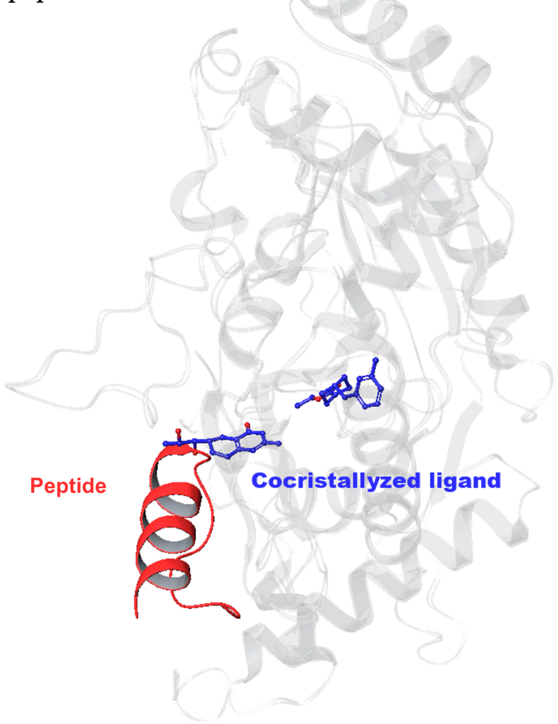

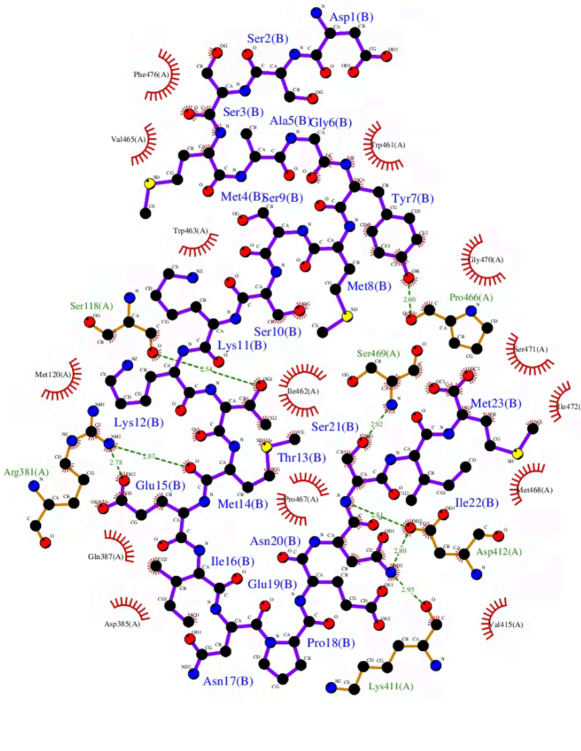

AIP peptide: NQKNLHKRYAYQIVLQTREMLR

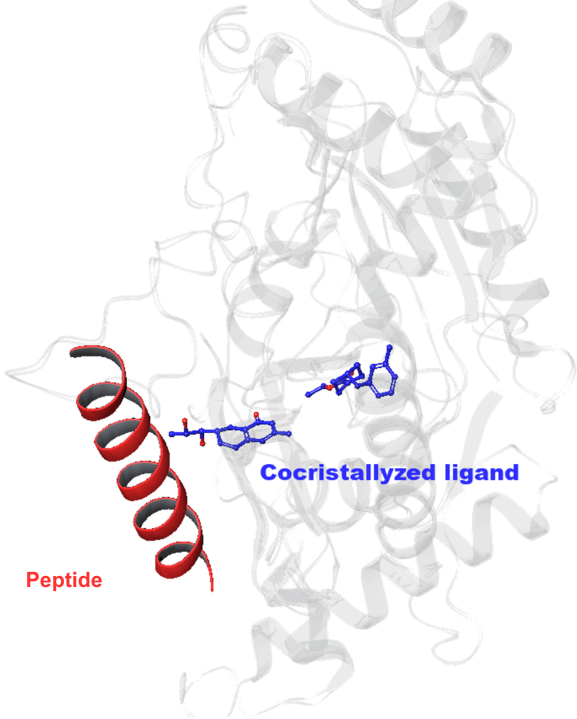

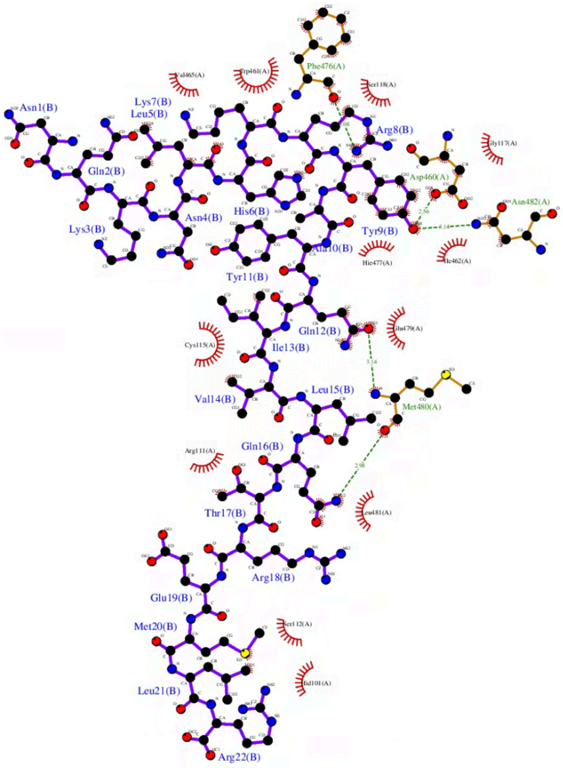

AIP peptide: DKRIFFTNKSYLPSQTPSGVIR

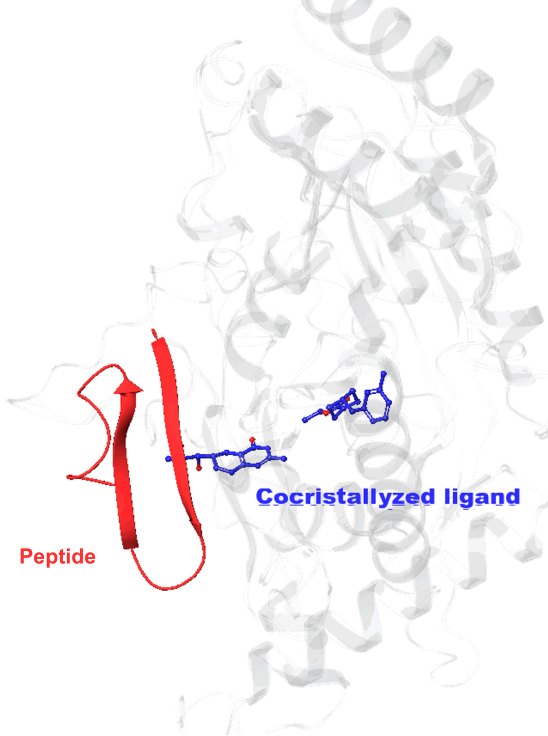

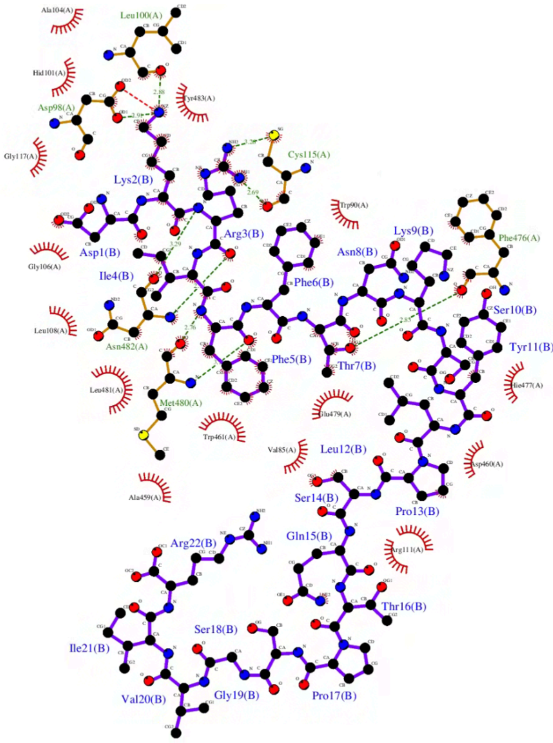

AIP peptide: DTQPPRLPTKAVRVTAEEVR

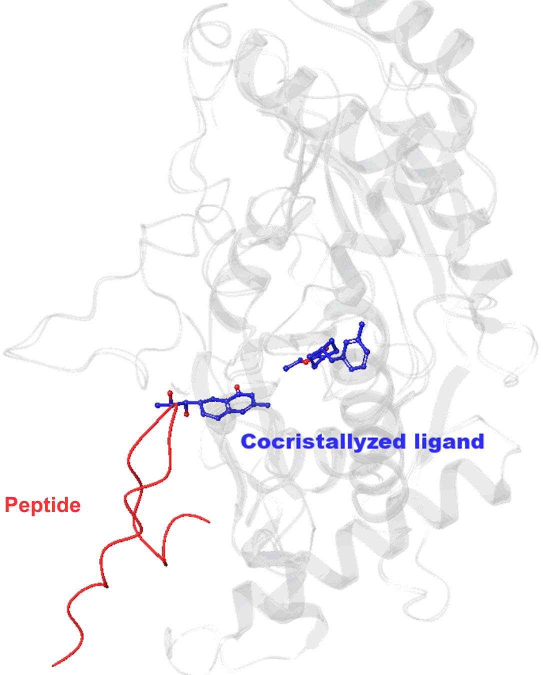

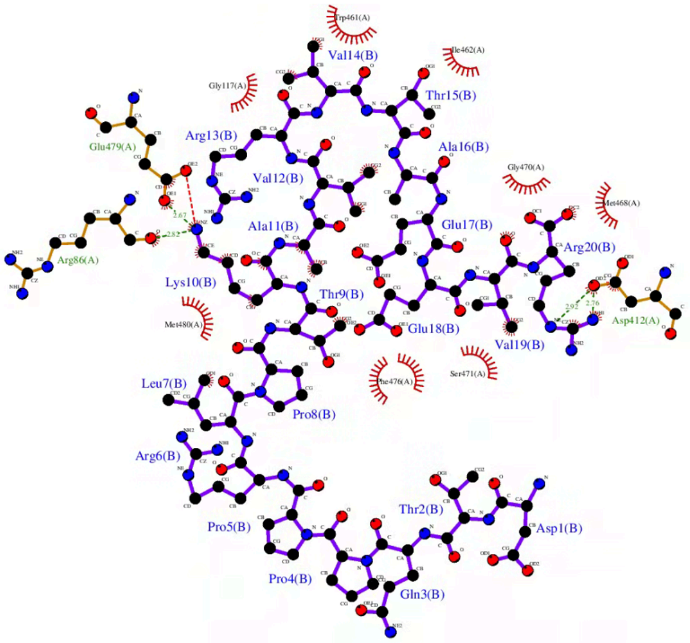

AIP peptide: DETPELMPLSHVLATKLGAR

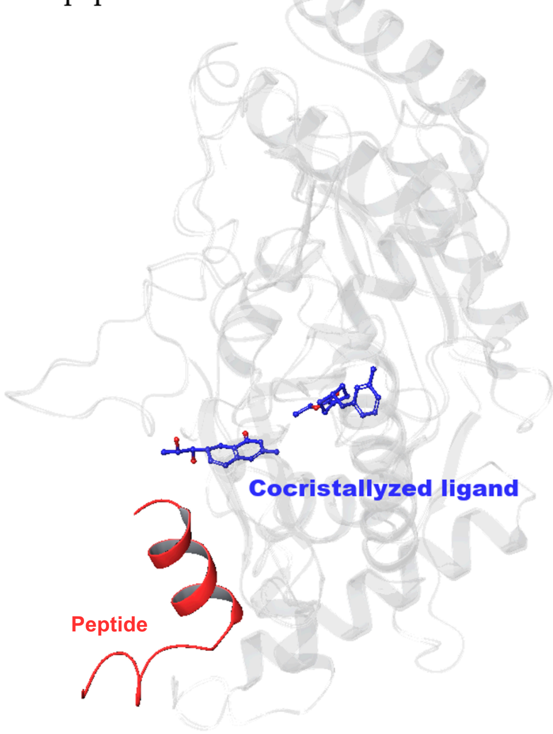

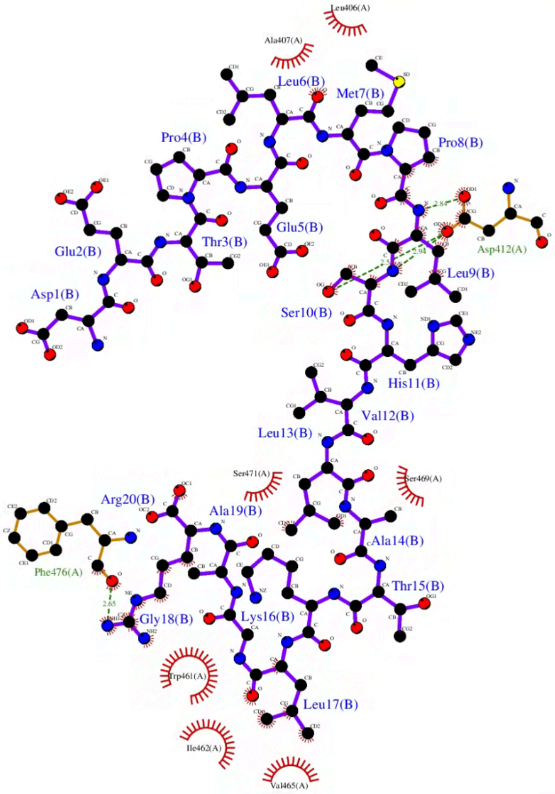

AIP peptide: SAILATPSGERTMTSEQMVY

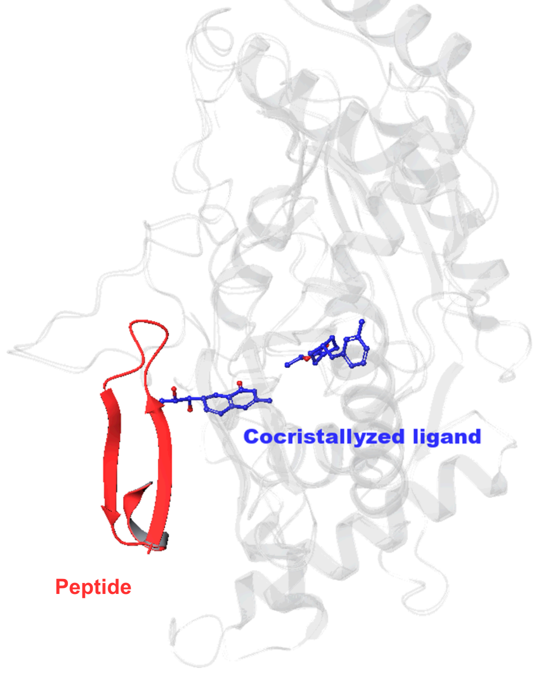

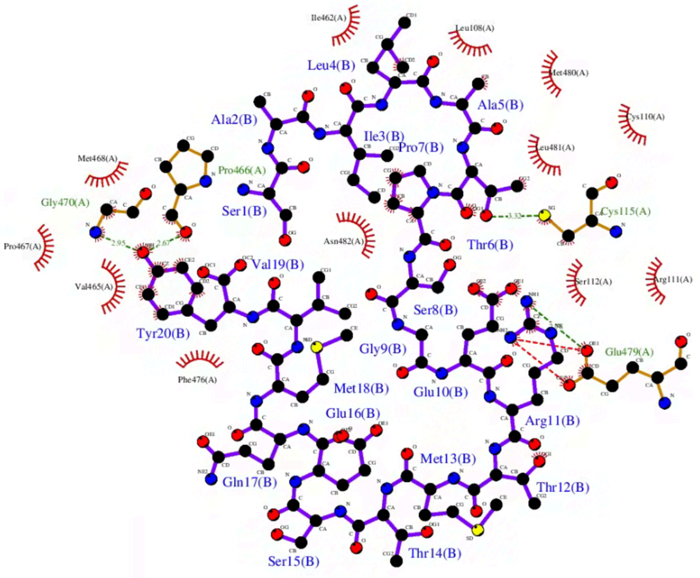

AIP peptide: CGYSMNSIEGAAVSTIHITPE

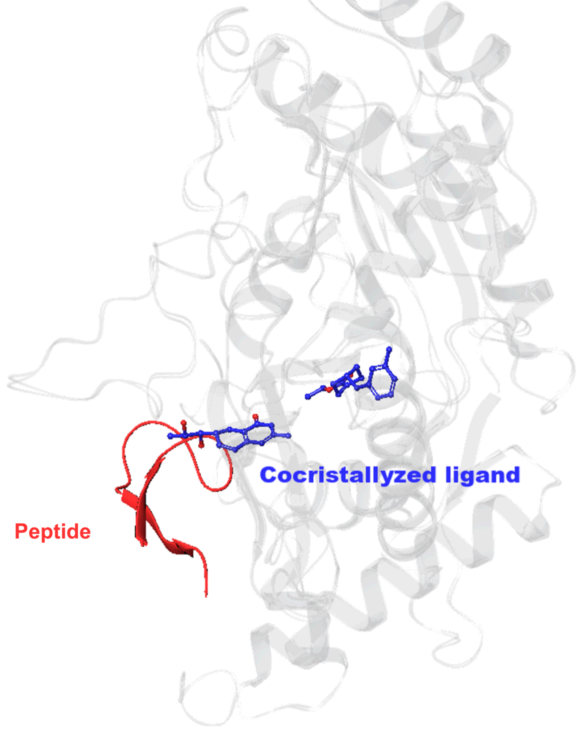

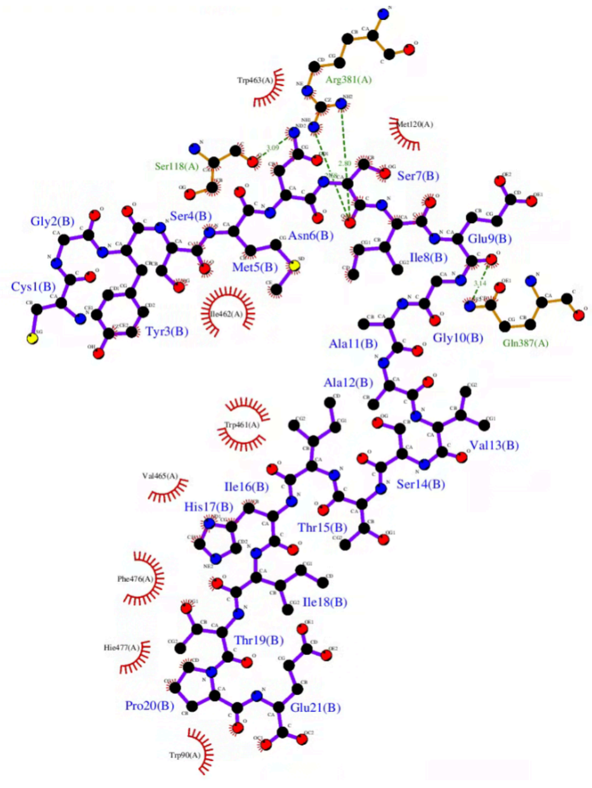

Figure S5.

Images of the best docking poses of AMP peptides in complex with UDP-N-acetylmuramoyl-L-alanyl-D-glutamate-L-lysine ligase (PDB ID: 4C12) of *Staphylococcus aureus* overlayed over co-crystallized ligand (PDB ID: 4C12), and LigPlot+ analysis. The amino acid residues involved in hydrophobic interactions are represented by red arcs, while hydrogen bonds are depicted as green dashed lines with their bond lengths specified.

AMP peptide: DGPNASYITPAAL

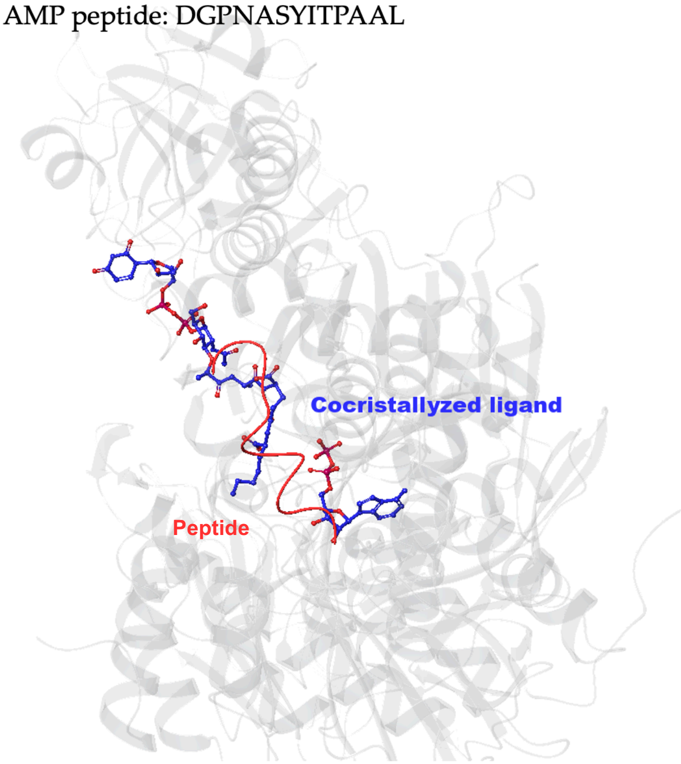

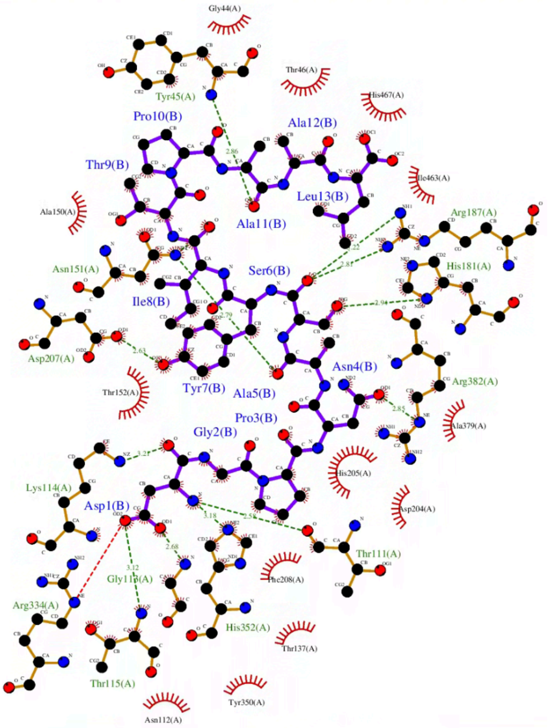

AMP peptide: DFGWGNPIFGGILKAISFTSFGVSVKN

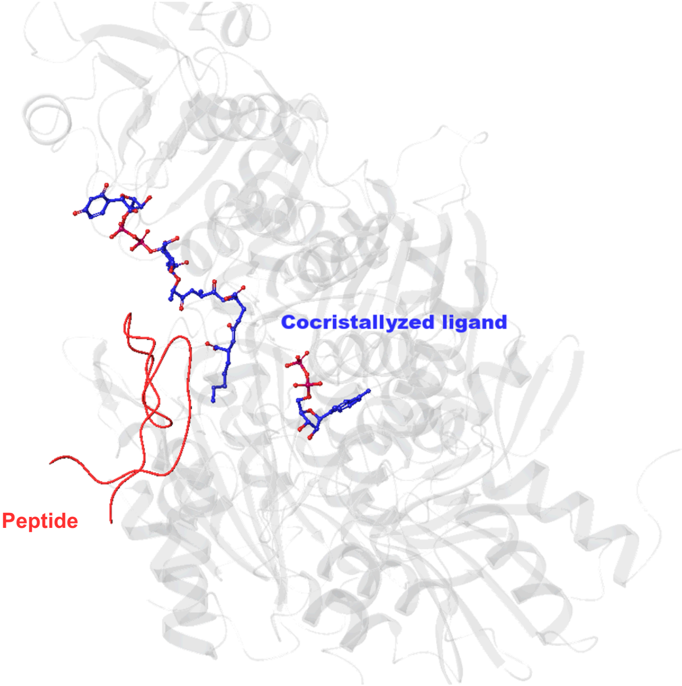

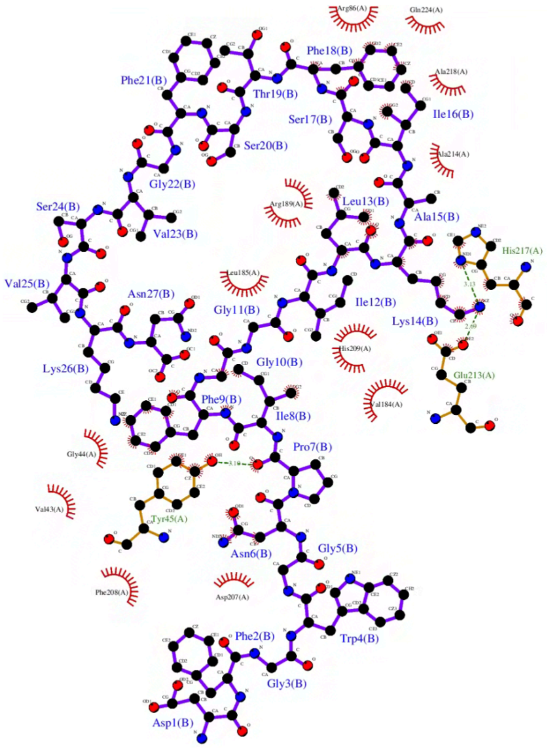

AMP peptide: DAGASKTYPQQAGTIRKGGHIVIKNR

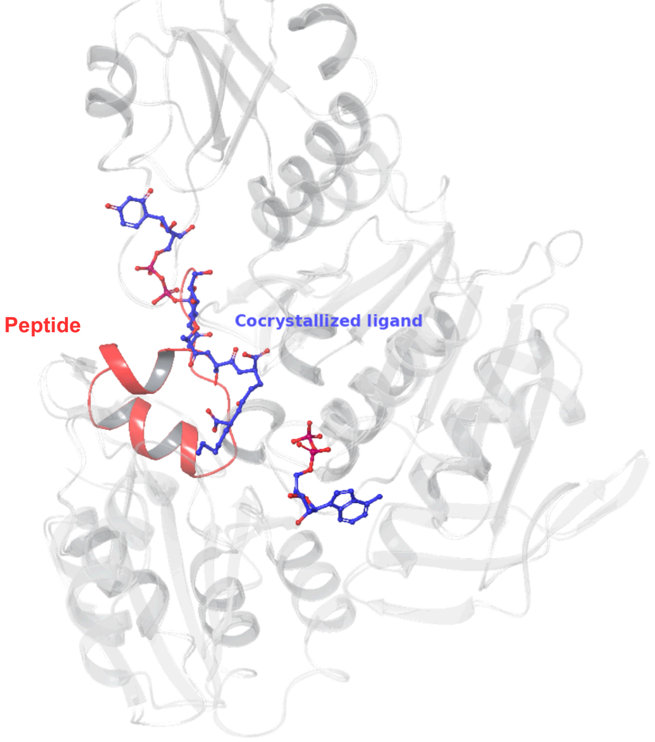

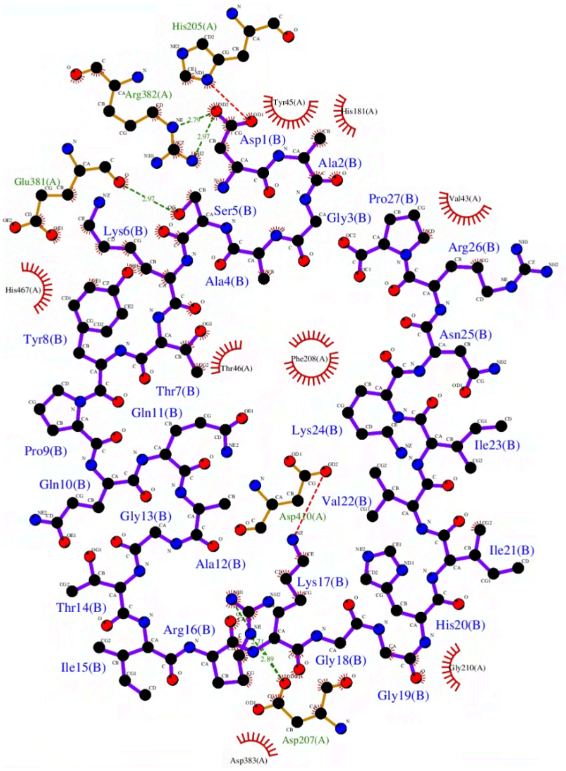

AMP peptide: DKVCVLSCGISTGLGASLNVAKP

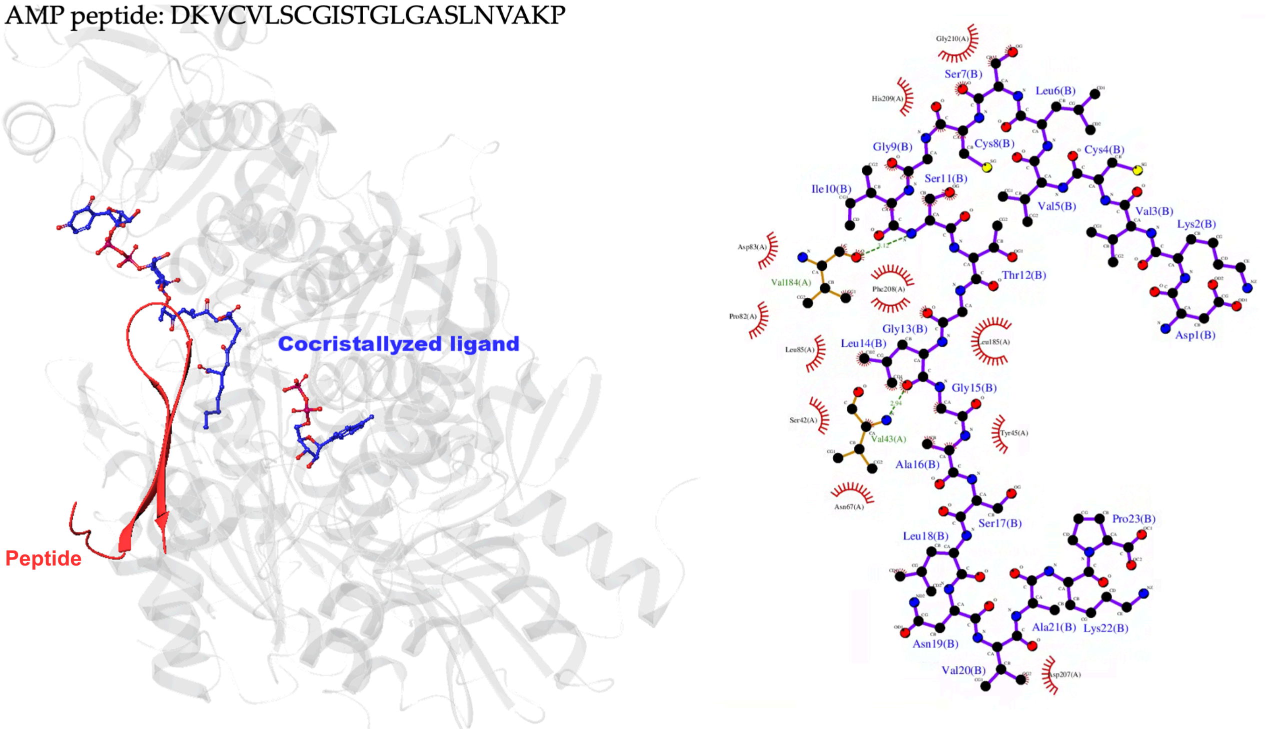

AMP peptide: DFLIGNTSTGYCAGGCAAIV

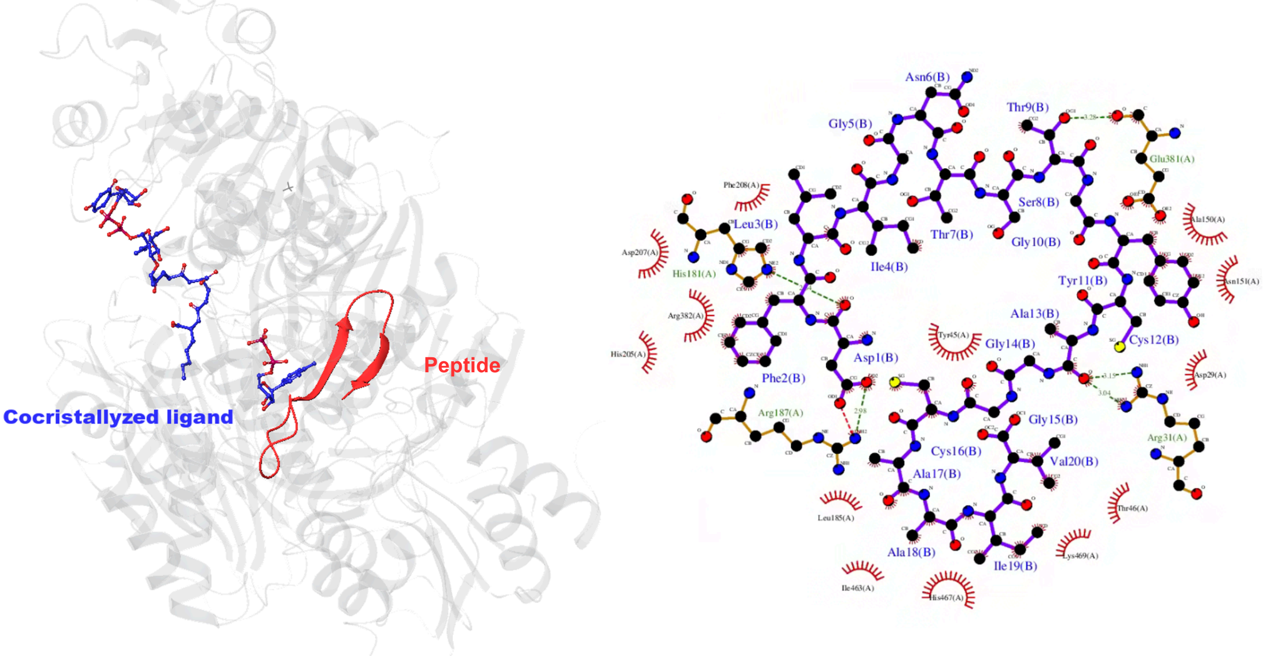

Figure S6.

Images of the best docking poses of AMP peptides in complex with Penicillin-Binding Protein 1a (PDB ID: 3QDL) of *Helicobacter pylori* overlayed over co-crystallized ligand (PDB ID: 3QDL), and LigPlot+ analysis. The amino acid residues involved in hydrophobic interactions are represented by red arcs, while hydrogen bonds are depicted as green dashed lines with their bond lengths specified.

AMP peptide: DAGASKTYPQQAGTIRKGGHIVIKNRP

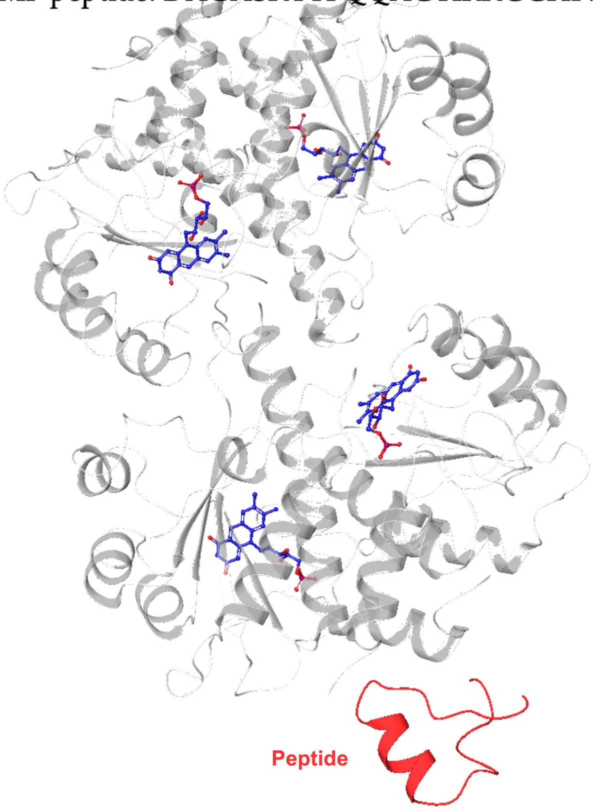

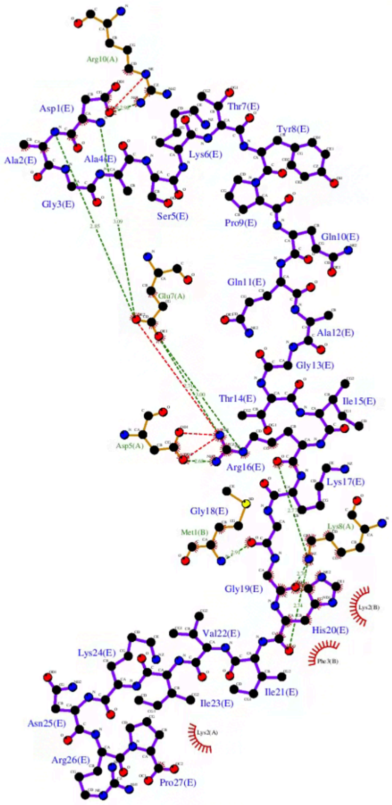

Figure S7.

Images of the best docking poses of AMP peptides in complex with Metallo-beta-lactamase type 2 (PDB ID: 6EW3) of *Pseudomonas aeruginosa* overlayed over co-crystallized ligand (PDB ID: 6EW3), and LigPlot+ analysis. The amino acid residues involved in hydrophobic interactions are represented by red arcs, while hydrogen bonds are depicted as green dashed lines with their bond lengths specified.

AMP peptide: DHVGFSCSTSGGAASRGILGPFGVIVIA

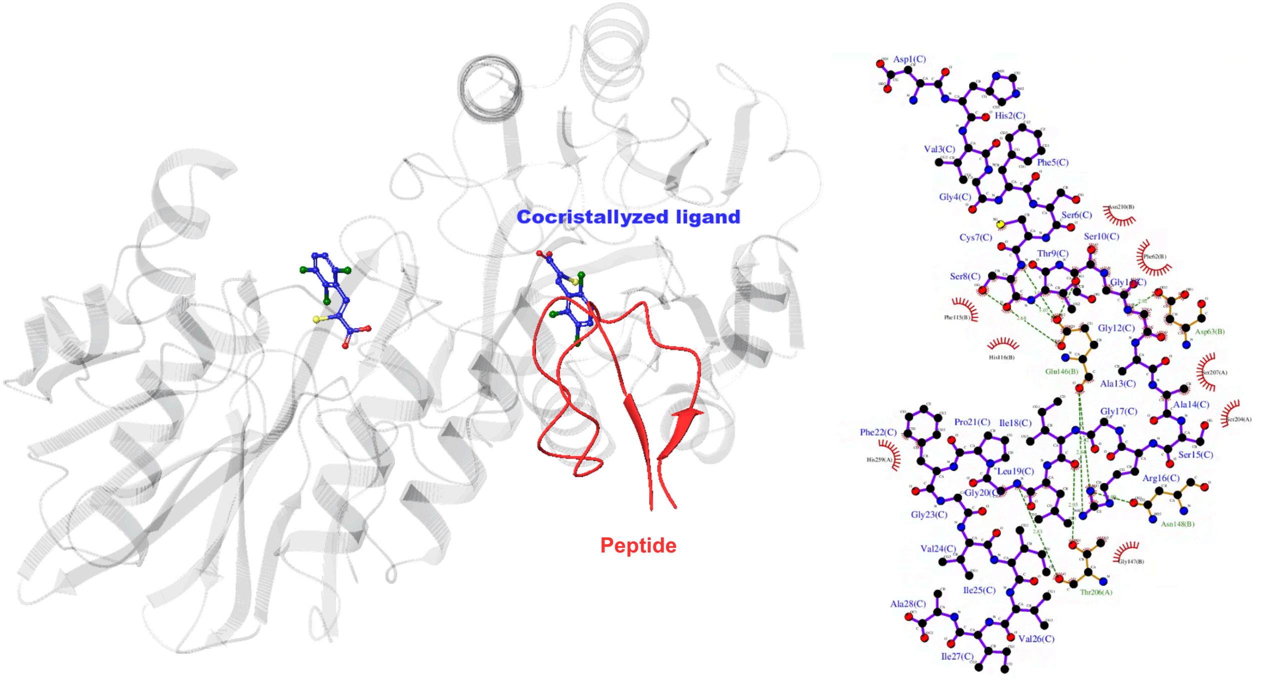

AMP peptide: DFLIGNTSTGYCAGGCAAIV

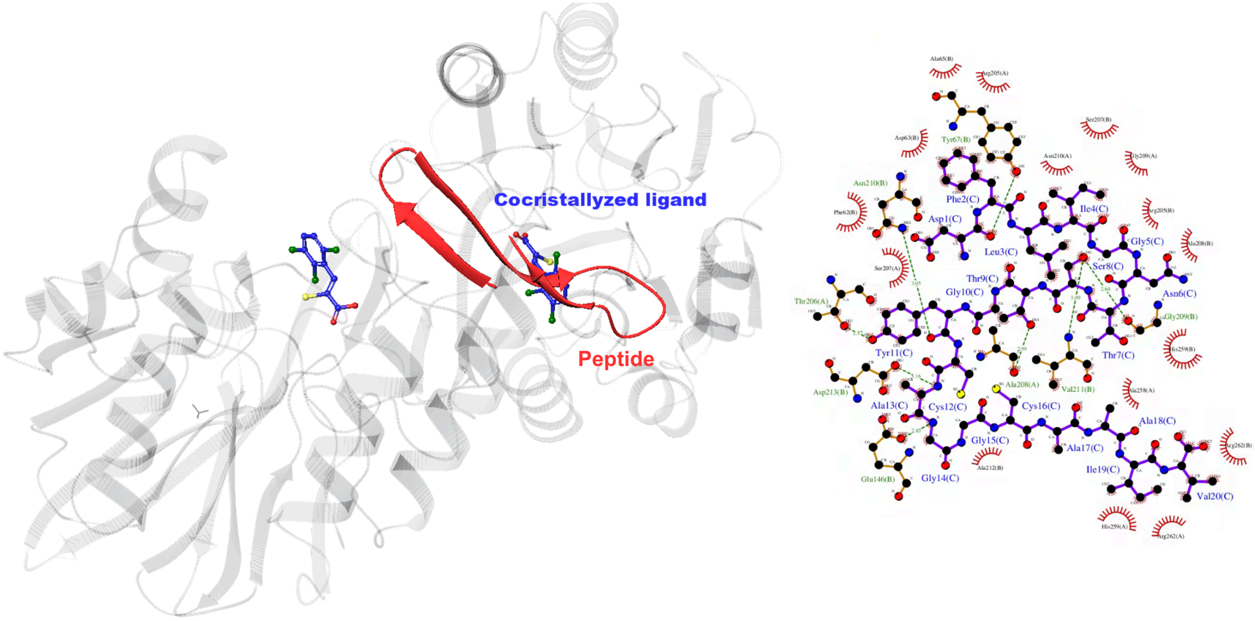

AMP peptide: DAGASKTYPQQAGTIRKNGYIVIKGRP

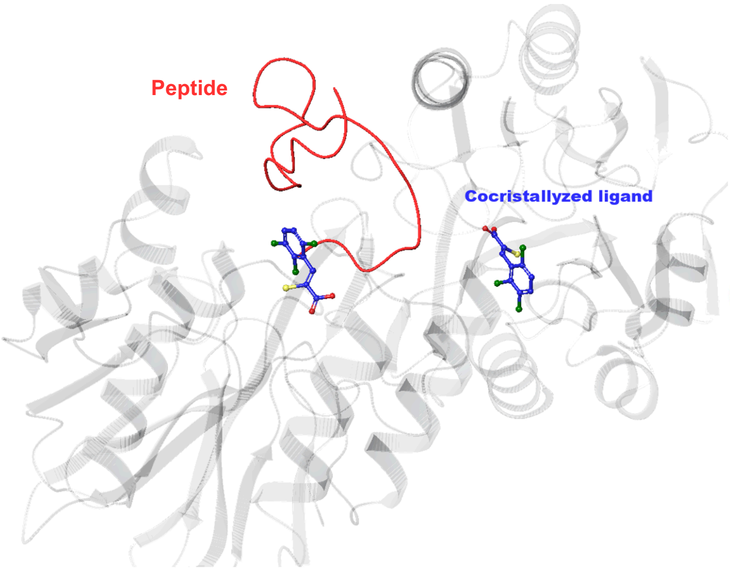

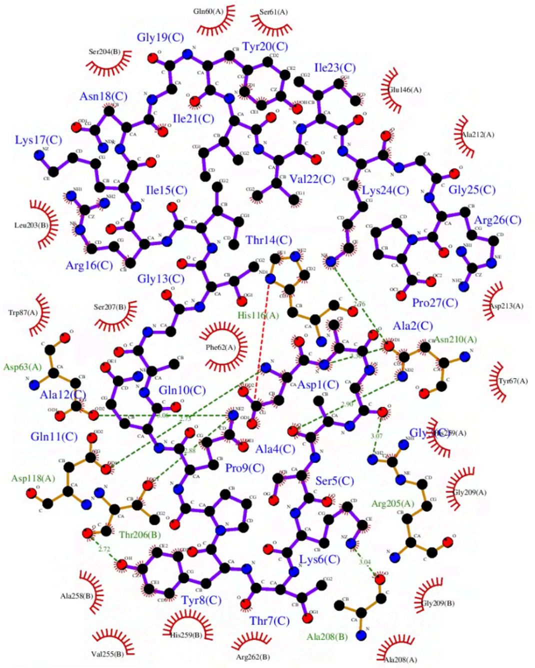

AMP peptide: DKVCVLSCGISTGLGASLNVAKP

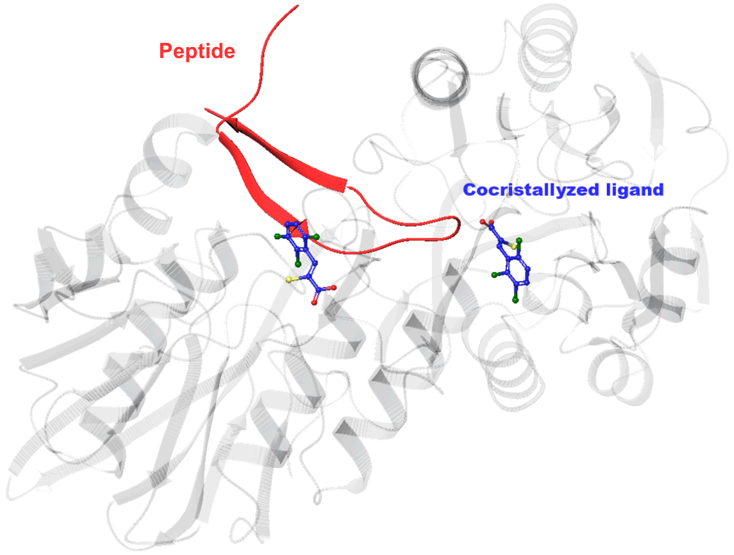

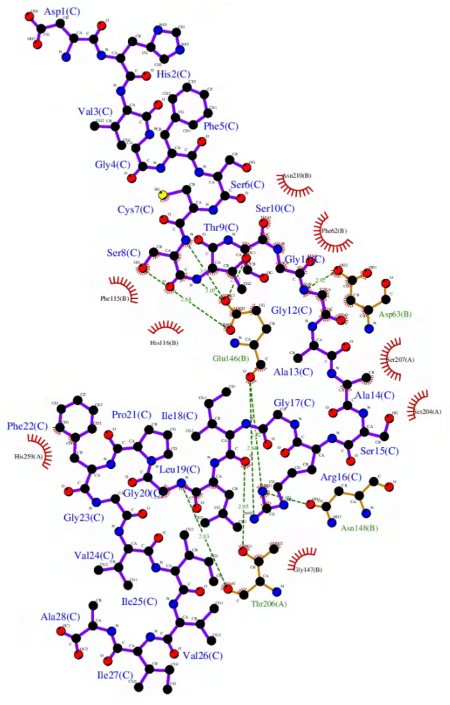

AMP peptide: DAGASKTYPQQAGTIRKGGHIVIKNRP

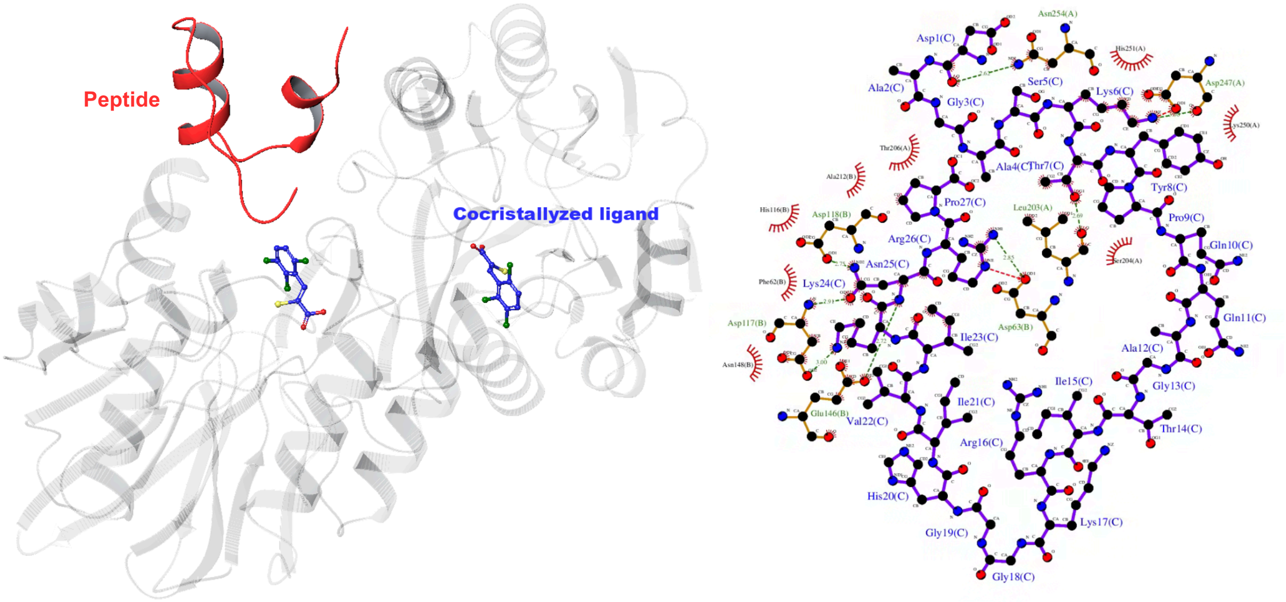

Figure S8.

Images of the best docking poses of AMP peptides in complex with Streptomycin 3″-adenylyltransferase (PDB ID: 6FZB) of *Salmonella enterica* overlayed over co-crystallized ligand (PDB ID: 6FZB), and LigPlot+ analysis. The amino acid residues involved in hydrophobic interactions are represented by red arcs, while hydrogen bonds are depicted as green dashed lines with their bond lengths specified.

AMP peptide: DHVGFSCSTSGGAASRGILGPFGVIVIA

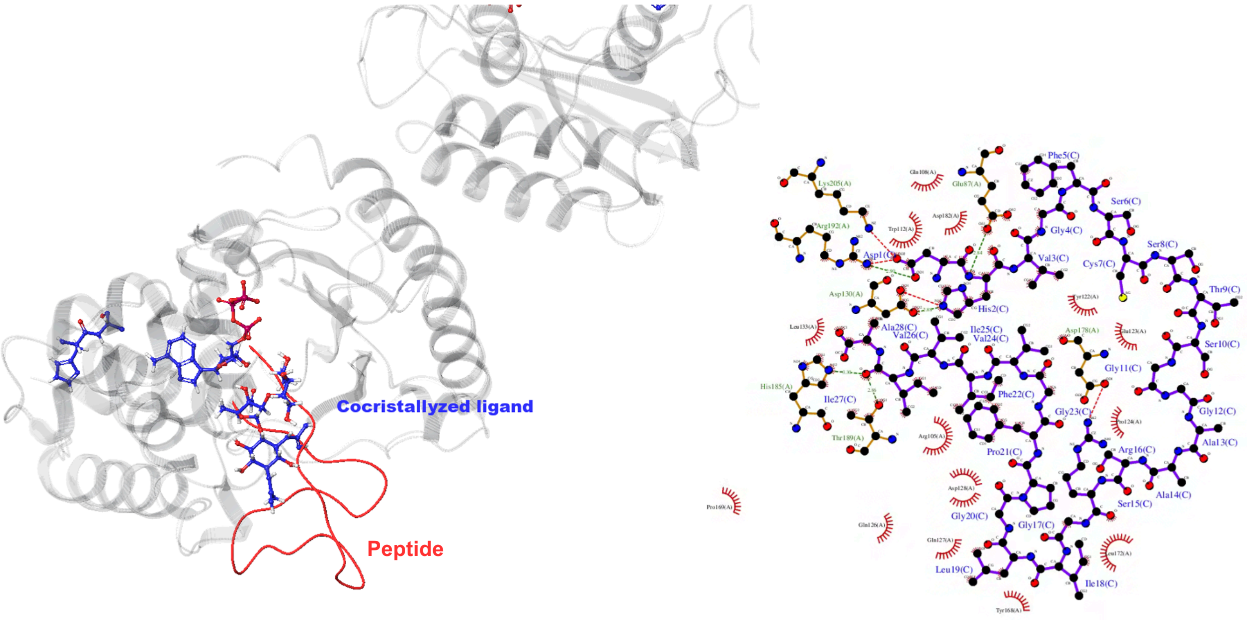

AMP peptide: DGPNASYITPAAL

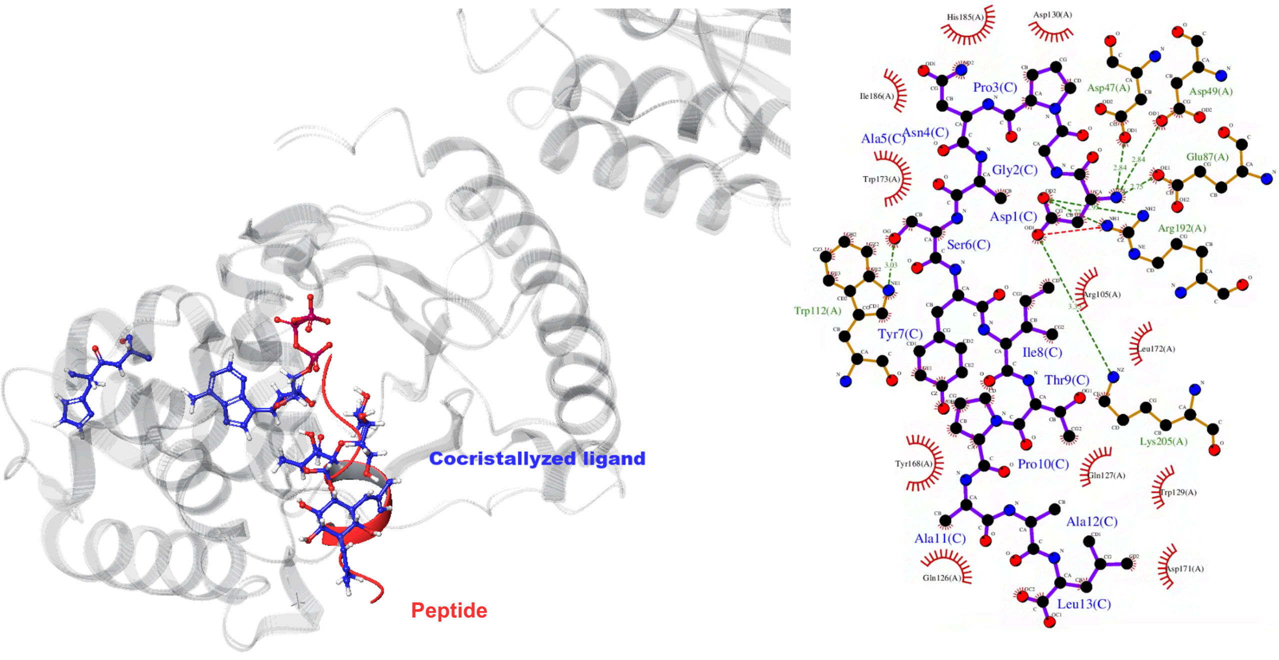

AMP peptide: DFLIGNTSTGYCAGGCAAIV

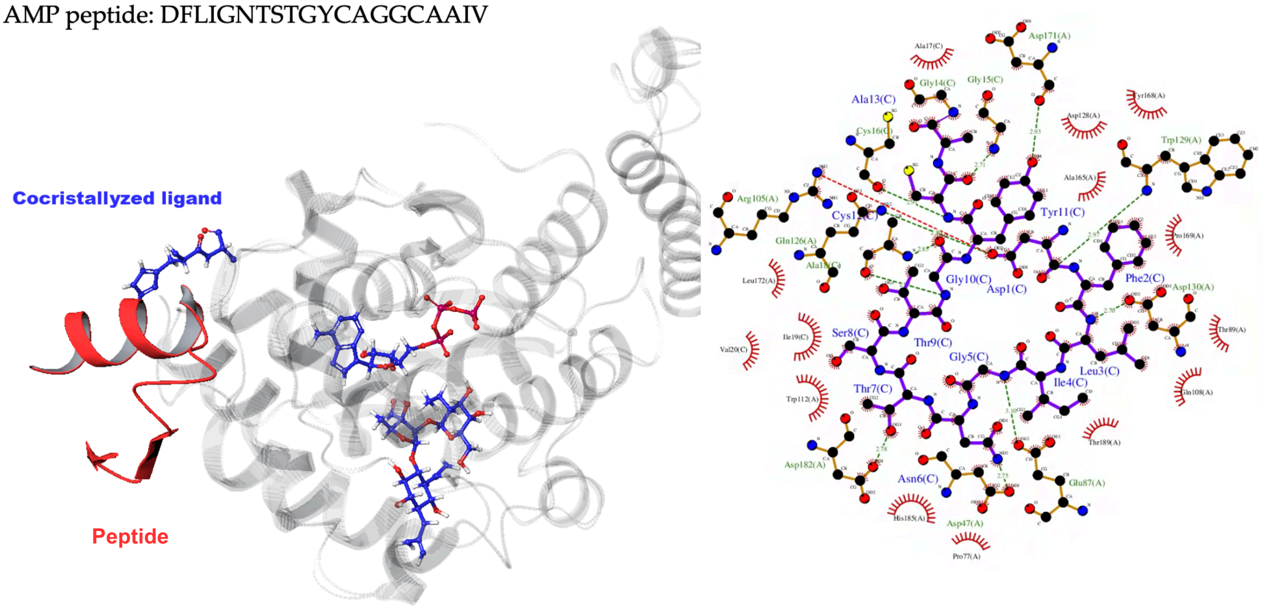

AMP peptide: DKVCVLSCGISTGLGASLNVAKP

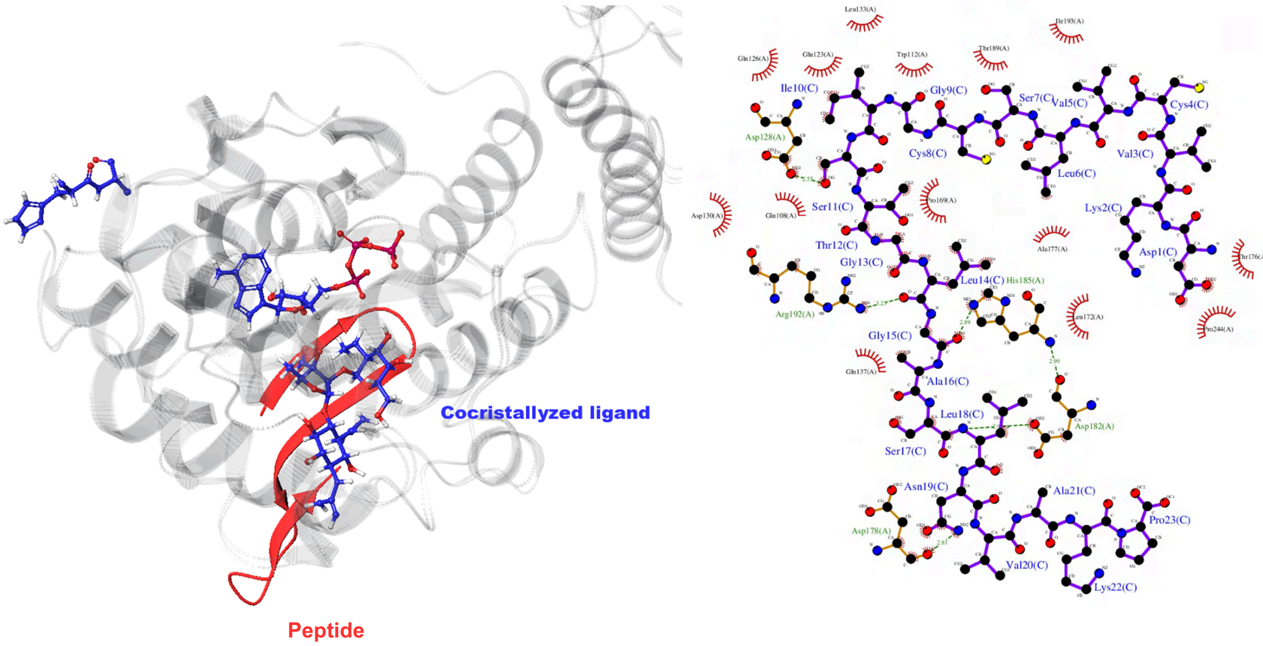

Figure S9.

Images of the best docking poses of AMP peptides in complex with 3UDF of *Acinetobacter baumanii* overlayed over co-crystallized ligand (PDB ID: 3UDI), and LigPlot+ analysis. The amino acid residues involved in hydrophobic interactions are represented by red arcs, while hydrogen bonds are depicted as green dashed lines with their bond lengths specified.

AMP peptide: DKVCVLSCGISTGLGASLNVAKP

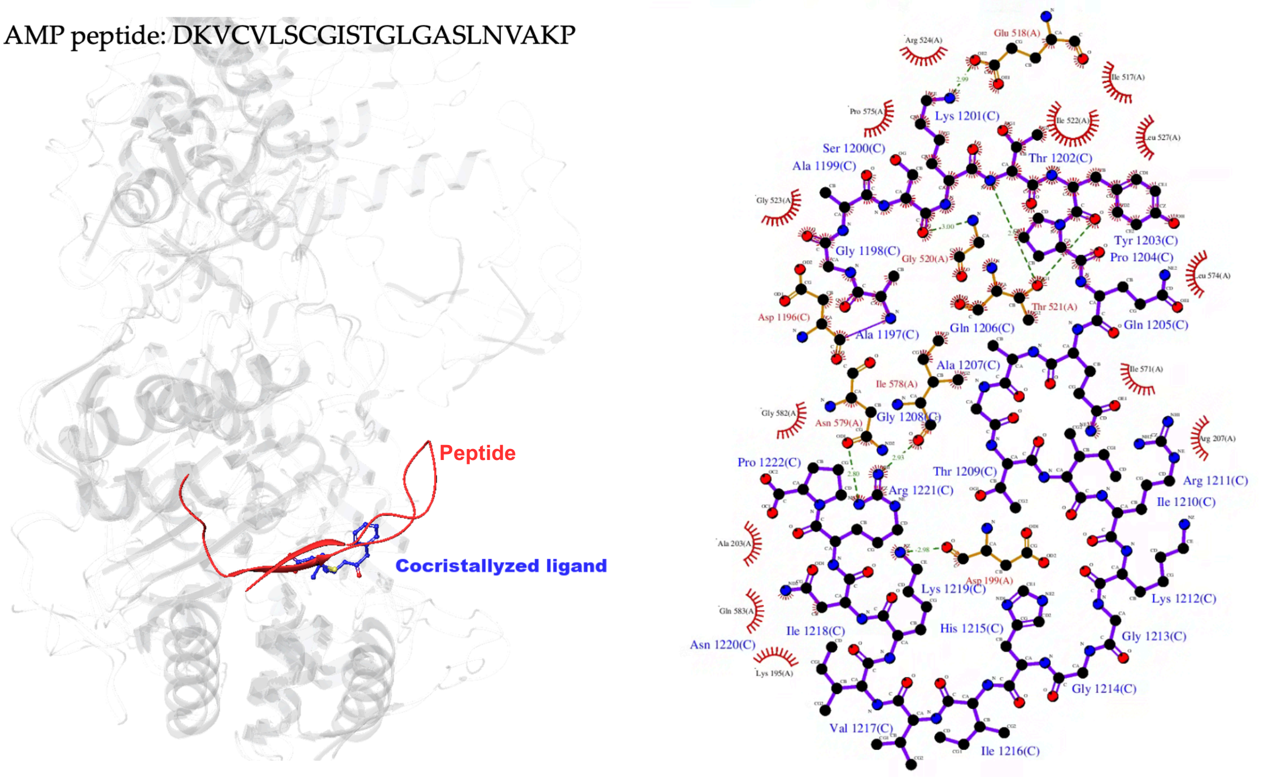

AMP peptide: DAGASKTYPQQAGTIRKGGHIVIKNRP

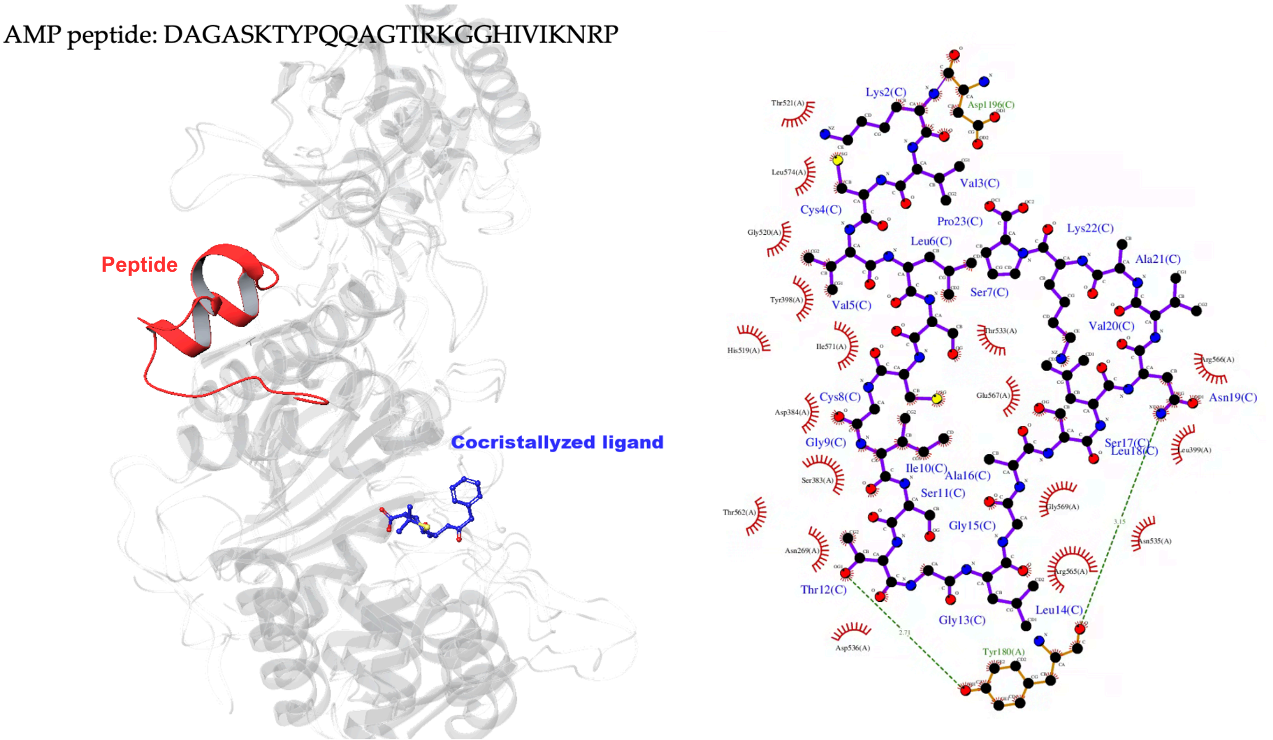

Supplement: Supplementary file 1 [file biomolecules-14-00930-s001.zip › Supplementary_file_Biomolecules-3111576.pdf]
